# Bifunctional Design of Ferroelectric‐Order and Band‐Engineering in Cu:KTN Crystal for Extended Self‐Powered Photoelectric Response

**DOI:** 10.1002/advs.202412877

**Published:** 2024-12-17

**Authors:** Yaqian Wang, Yabo Wu, Fei Liang, Xuping Wang, Haohai Yu, Huaijin Zhang, Yicheng Wu

**Affiliations:** ^1^ State Key Laboratory of Crystal Materials and Institute of Crystal Materials Shandong University Jinan 250100 China; ^2^ Xinjiang Technical Institute of Physics and Chemistry Chinese Academy of Sciences Urumqi 830011 China; ^3^ Advanced Materials Institute Qilu University of Technology (Shandong Academy of Sciences) Jinan 250014 China

**Keywords:** ferroelectrics, KTa_1−_ *_x_* Nb*_x_* O_3_ perovskite crystal, photodetector, self‐powered responsivity

## Abstract

Photoelectric conversion in ferroelectric crystals can support many important applications in modern on‐chip technology, but suffering from two problems, low responsive current and narrow responsive range. Especially, wide‐gap ferroelectric oxides are only active at short‐wavelength ultraviolet region with weak photocurrent at nanoampere levels. Here, a bifunctional design strategy of ferroelectric‐order and electronic‐band to improve the photocurrent and extend the responsive range simultaneously, is proposed. In a Cu‐doped KTa_1−_ *_x_* Nb*_x_* O_3_ (KTN) perovskite crystal, a conductive channel is constructed by “head‐to‐head” ferroelectric domains, associated with the emergence of micrometer‐scale supercells. In addition, the introduction of Cu^+^ ion can induce defect levels, thus extending the responsive range beyond the inherent absorption of pure KTN. Through rational device optimization, a record self‐powered responsivity of 5.23 mA W^−1^ is realized in Cu:KTN photodetector, which is two orders of magnitude higher than undoped KTN crystal. The temperature‐dependent light diffraction and photocurrent show that the ferroelectric‐order is dominated in this photoresponse behavior. Moreover, Cu:KTN detector is active in the broadband range from 390 to 1030 nm, covering ultraviolet, visible, and near‐infrared regions. This work provides an effective method for the design of next‐generation self‐powered photodetectors with ultrahigh responsivity and ultrawide responsive range.

## Graphical abstract

This work proposes a bifunctional design strategy of ferroelectric‐order and electronic‐band to improve the photocurrent and extend the responsive range simultaneously. In a Cu‐doped KTa_1−_ *_x_* Nb*_x_* O_3_ perovskite crystal, a conductive channel is constructed by “head‐to‐head” ferroelectric domains, associated with the emergence of micrometer‐scale supercells, which is helpful to improve the photocurrent.

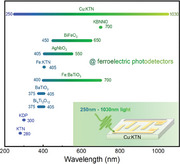

## 1 Introduction

Ferroelectric crystal is one of the excellent multifunctional materials applied in modern scientific technology.^[^ ^1^, ^2^, ^3^ ^]^ It holds a distinctive feature, that the electrical polarization can be easily switched by the external electrical field.^[^ ^4^ ^]^ As a result, ferroelectric crystals have been widely applied in many electronic devices,^[^ ^5^, ^6^, ^7^, ^8^, ^9^ ^]^ such as non‐volatile memory,^[^ ^10^ ^]^ multilayer ceramic capacitors,^[^ ^11^ ^]^ photovoltaic,^[^ ^12^ ^]^ and optical frequency converters.^[^ ^13^ ^]^ In the optical community, ferroelectric crystals have become necessary for many scenarios with optoelectronic applications. For example, periodic poled LiNbO_3_ and LiTaO_3_ can support broadband and efficient harmonic‐wave generation.^[^ ^14^, ^15^ ^]^ Transparent PMN‐PT crystal induced by alternating current (a.c.) poling has been invented and can be utilized in medical imaging and energy‐harvesting touch screens.^[^ ^16^ ^]^ Rhombohedral PIN‐PMN‐PT crystal with a giant electro‐optic coefficient is reported for the fabrication of ultracompact EO Q‐switches with a low driving voltage.^[^ ^17^ ^]^ At present, designing novel ferroelectric materials and devices for optical applications has always been a hot topic in the scientific community.

The optical functions of ferroelectric crystals originate from the ferroelectric domains, as well as their response behavior to external electromagnetic fields.^[^ ^18^, ^19^, ^20^, ^21^ ^]^ In terms of light–matter interaction, some intriguing domain super‐structures have been designed in ferroelectric crystals for the realization of long‐term desired properties. For example, 3D nonlinear photonic crystals have been fabricated to control nonlinear interacting light waves in 3D configuration. Ferroelectric domain inversion in the nanoscale is also demonstrated in LiNbO_3_ by the non‐reciprocal laser‐writing technique,^[^ ^13^ ^]^ which has potential applications in high‐efficiency photoelectric conversion. Moreover, the interaction of ferroelectricity and light enables ferroelectrics to detect optical signals at zero‐bias voltage, namely, self‐powered photodetector.^[^ ^22^ ^]^ This case is first discovered by Fridkin as a bulk photovoltaic effect.^[^ ^23^ ^]^ This photocurrent is driven by the internal electric field constructed by ferroelectric domains, to separate the photo‐induced carriers in bulk crystal. Compared to the traditional p–n junction or Schottky junction, the open‐circuit voltage of bulk photovoltaic materials can break the limit of the intrinsic band gap and display high photoelectric conversion efficiency.^[^ ^24^, ^25^ ^]^ To the best of our knowledge, this phenomenon has been observed in many ferroelectric crystals, for example, LiNbO_3_, BiFeO_3_, KH_2_PO_4_, and BaTiO_3_, with various ferroelectric‐domain patterns.^[^ ^26^, ^27^, ^28^, ^29^, ^30^, ^31^, ^32^ ^]^

Despite of great potential of self‐powered ferroelectric photodetectors, however, their photocurrent and responsivity are still relatively low (usually below 1 µA W^−1^).^[^ ^31^, ^32^, ^33^, ^34^, ^35^, ^36^, ^37^ ^]^ This low current is assigned to the inflexible directions of ferroelectric domains in high‐*T*_c_ ferroelectrics. In addition, most ferroelectric oxides have a large forbidden gap of more than 2.5 eV,^[^ ^12^, ^38^, ^39^ ^]^ thereby leading to a narrow responsive range in the ultraviolet region. These two obstacles hinder the actual applications of ferroelectric‐based photodetector. To improve the photo‐responsive performances and extend the photosensitive range concurrently, some modified methods including ion‐doping, defect‐engineering, thermal annealing, etc., have been utilized in some low‐dimensional photodetectors, such as BiFeO_3_ film,^[^ ^40^, ^41^ ^]^ LiNbO_3_ film,^[^ ^42^ ^]^ and BaTiO_3_‐based detectors.^[^ ^43^ ^]^ However, in bulk ferroelectric crystals with self‐powered properties, these studies are rarely reported. Therefore, at present, it is still a great challenge to make a high‐responsive self‐powered ferroelectric photodetector in a broadband spectral range.

In 2022, our group proposed an inversed design strategy to rearrange polar ferroelectric domains in the KTa_1−_ *_x_* Nb*_x_* O_3_ (KTN) crystal and improve its self‐powered responsivity significantly.^[^ ^19^ ^]^ A periodic electrical potential fluctuation can be conducted with high‐conductive channels at domain walls, acting as a “ferroelectric highway” for electrons and holes. In this work, we fabricate a high‐performance ferroelectric detector by Cu‐doped KTN crystal. A bifunctional design is applied in Cu:KTN, that a ferroelectric supercell at micrometer‐scale is built to separate photo‐induced carriers and the absorption edge is red‐shifted from ultraviolet to near‐infrared. As a result, Cu:KTN photodetector holds a high responsivity up to 5.23 mA W^−1^, which is two orders of magnitude higher than pure KTN single crystal and four orders of magnitude higher than BaTiO_3_ single crystal. Meanwhile, Cu:KTN detector is optical active in a broadband spectral range from 390 to 1030 nm, which is wider than most of the state‐of‐the‐art ferroelectric single‐crystal detectors. These results indicate that the performances of self‐powered ferroelectric detectors can be manipulated by the synergistic design of ferroelectric order and band engineering.

## 2 Results and Discussion

### 2.1 Electronic Band Structure Calculations of Cu:KTN Crystal

KTN crystal exhibits a typical ABO_3_ perovskite structure, which is composed of (Ta/Nb)O_6_ octahedra with co‐apex angles, and the central B position is alternately occupied by Ta and Nb atoms. The Curie temperature of KTN crystal can be easily adjusted by changing the Ta/Nb ratio.^[^ ^44^ ^]^ In general, the crystal phase of KTN crystal will change from cubic phase (*x* < 0.40) to tetragonal phase (0.40 < *x* < 0.56), to orthorhombic phase (*x* > 0.56) at room temperature.^[^ ^45^ ^]^ In the tetragonal ferroelectric phase, the metal ion at the B‐site can move along the four‐fold axis, which leads to the distorted (BO_6_) octahedron and the spontaneous polarization in KTN crystal. As a result, the electrostatic potential in KTN crystal is regulated by the ferroelectric polarization inside the crystal, thus affecting the separation and migration of photon‐induced carriers. In addition, the bandgap of oxide perovskites is generally larger than 3 eV.^[^ ^46^, ^47^ ^]^ In previous studies, the absorption edge of ferroelectric KTN crystals is located at 393 nm. Therefore, pure KTN crystals are only active at the short‐wavelength ultraviolet range, which hinders their self‐powered detection capacity in the visible and near‐infrared regions.

Here, we propose a bifunctional strategy for increasing the photocurrent of ferroelectric crystal and extending the responsive range concurrently. In previous studies on photorefractive KTN, Fe:KTN, Mn:KTN, and Cu:KTN crystals have been reported for many years.^[^ ^48^, ^49^ ^]^ However, the photo‐response of Fe:KTN crystal in the ultraviolet region become deteriorated owing to its complex ferroelectric order in the orthorhombic phase.^[^ ^50^ ^]^ In addition, the absorption range of Fe:KTN is extended to 600 nm,^[^ ^51^ ^]^ which is insufficient for the broadband response. In comparison, Cu:KTN maintains the tetragonal phase and displays some anomalous ferroelectric domain structures. Therefore, we choose Cu as the doping ion in this work.

**Figure 1:**
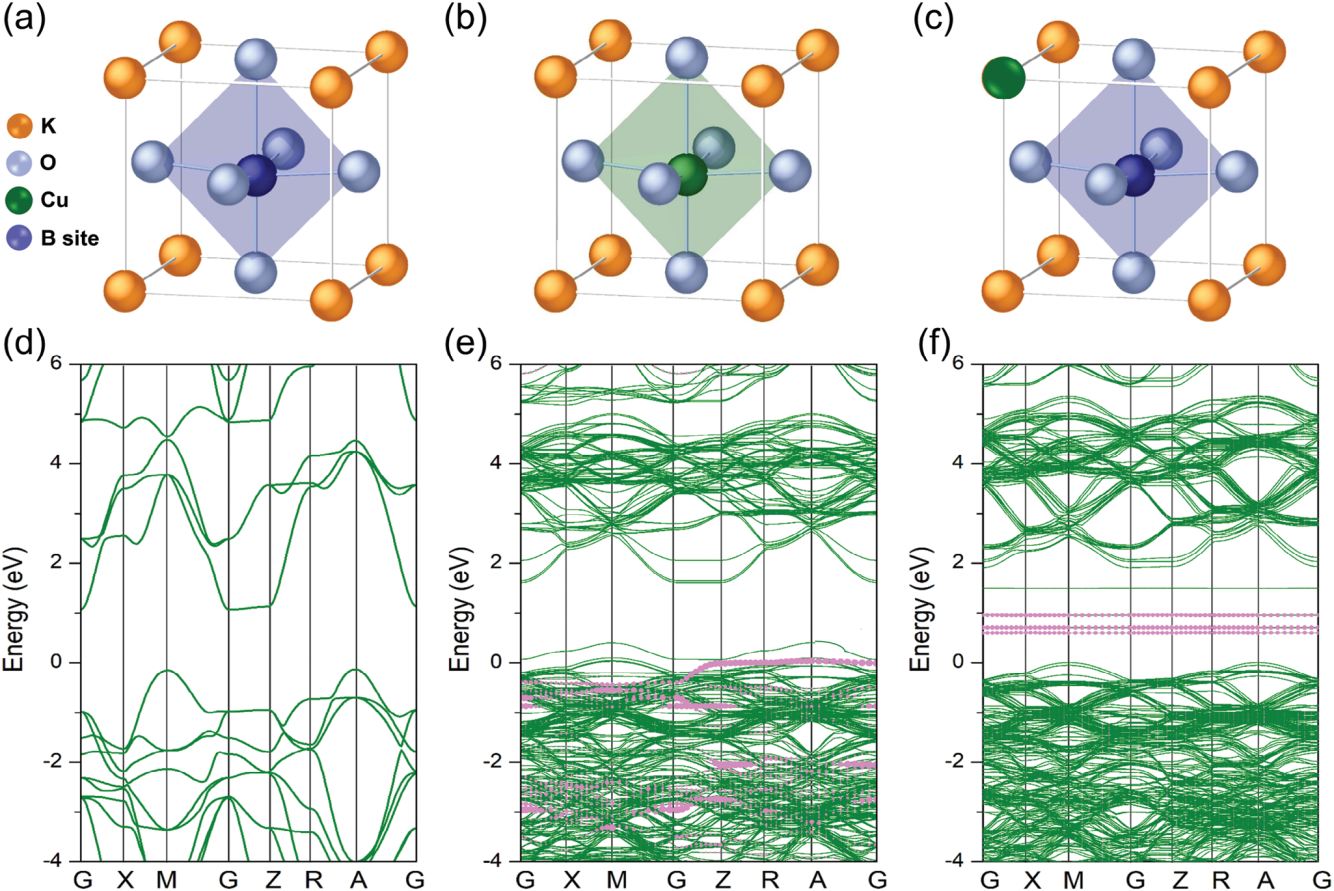
Crystal structure and density functional theory calculation of Cu substitution at different sites of KTN crystal. a) Crystal structure of pure KTN crystal. b) Crystal structure of Cu:KTN crystal with substituted at B site. c) Crystal structure of Cu:KTN crystal with substituted at A site. d) Electronic band structure of KTN crystal. e) Electronic band structure of Cu:KTN crystal with substituted at B site. f) Electronic band structure of Cu:KTN crystal with substituted at A site. The pink bands in (e) and (f) are defect levels of doped Cu^+^ ions.

**Figure** **1** depicts the crystal structure and electron band structure of KTN and Cu:KTN crystals. Here, we use tetragonal KTN to simulate the disordered Ta–Nb solid solutions. The ionic radius of Nb^5+^, Ta^5+^, K^+^, and Cu^+^ ion is 0.64, 0.64, 1.38, and 0.77 Å, respectively. Two possible substitution sites, K^+^ ion at the A site and Nb^5+^ ion at the B site, are considered. Pure KTN crystal is an indirect‐gap semiconductor, with the maximum valence band at M point and the minimum conduction band at G point. When Nb^5+^ ion is replaced by Cu^+^ ion, there are some mixed defect levels located at the upper valence band. When the K^+^ ion is replaced by the Cu^+^ ion, there are some defect levels at the conduction band from doping Cu^+^ ion. In addition, the fluorescent spectrum of Cu:KTN also demonstrates that the valence state of Cu ion is +1 (Figure S1, Supporting Information), which is the same as K^+^ ion at the A‐site. Therefore, taking ionic radius and valence state into account, Cu‐doping at the K^+^ site should be more possible in Cu:KTN. Benefitting from the emergence of defect levels, the absorption range of Cu:KTN will extend from ultraviolet to infrared region, which is favorable for designing a broadband self‐powered photodetector.

### 2.2 Crystal Growth and Characterization of Cu:KTN

**Figure 2:**
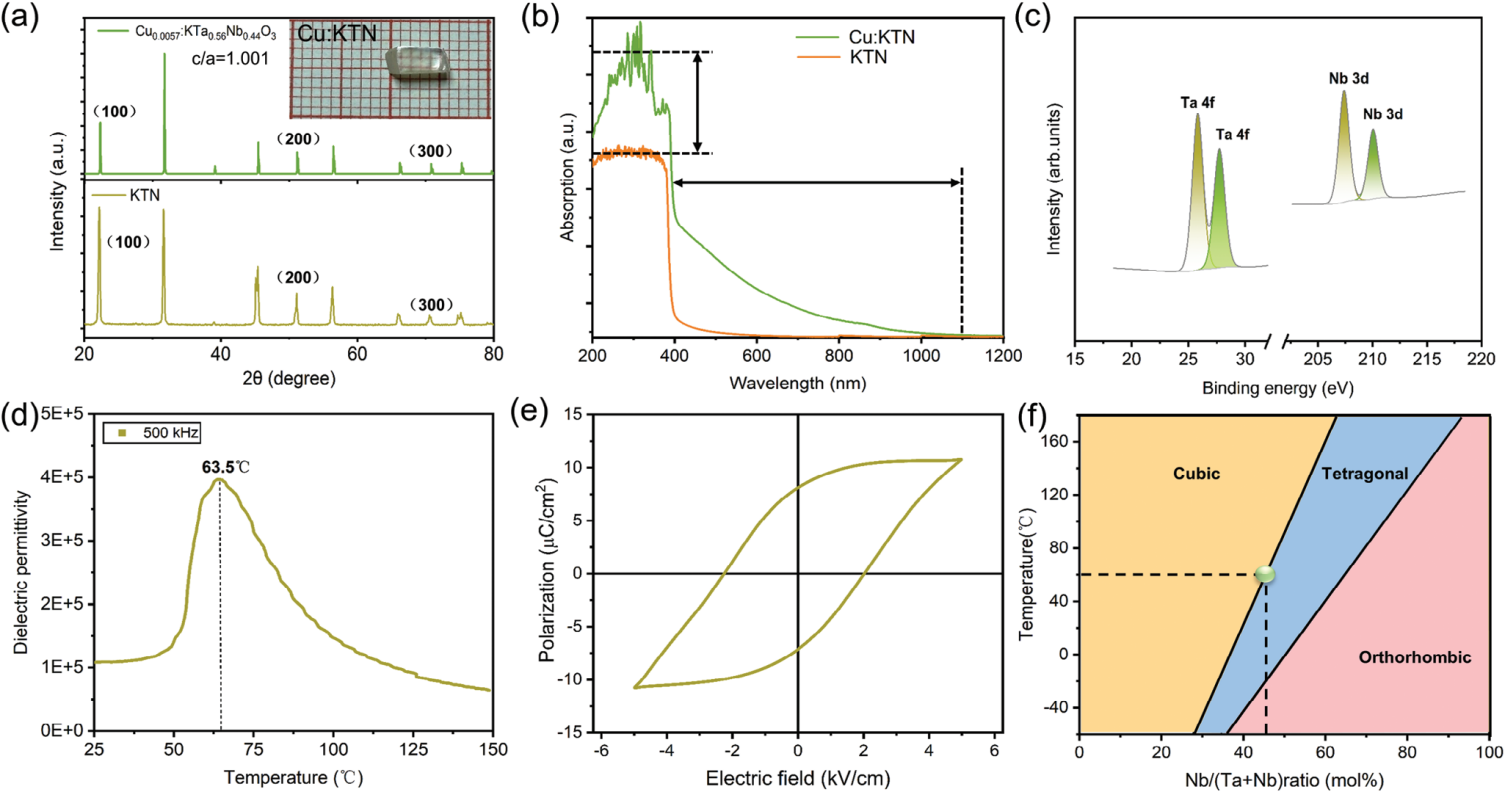
Characterization of Cu:KTN crystal. a) X‐ray diffraction (XRD) pattern comparison between Cu:KTN and KTN. The insert graph is Cu:KTN crystal. b) The absorption spectrum of Cu:KTN and KTN crystal. c) X‐ray photoelectron spectroscopy (XPS) of Cu:KTN crystal. d) Temperature‐dependent dielectric function spectrum of Cu:KTN crystal. e) Hysteresis loop of Cu:KTN crystal. f) Phase diagram of KTN crystal.

High‐quality Cu:KTN crystals were grown by the off‐center top‐seeded solution growth (TSSG) method. The crystal growth method is given in the Experimental Section. **Figure** **2**a shows the XRD patterns of Cu:KTN and KTN crystals. All the diffraction lines of Cu:KTN are consistent with the KTN, indicating that Cu:KTN crystal has a tetragonal perovskite structure and there are no impurity phases with Cu‐doping. By solving the single crystal structure, the *c*/*a* ratio of Cu:KTN and pure KTN crystal is 1.001 and 1.0036, respectively. In general, the greater the *c*/*a* ratio deviation from 1, the stronger the lattice distortion of KTN. Therefore, we can reasonably infer that Cu ion doping alleviates the lattice distortion of KTN to a certain extent.

In addition, the absorption spectra of Cu:KTN crystal and KTN crystal were measured (Figure 2b). The absorption of UV light is significantly enhanced after Cu‐doping, and the absorption cut‐off edge redshifts from 390 to 1100 nm, covering the entire visible region and extending to the near‐infrared band. According to X‐ray photoelectron spectroscopy (XPS) in Figure 2c, the Ta/Nb ratio of Cu:KTN crystal is 0.56/0.44, indicating that it is a tetragonal phase at room temperature. Moreover, the inductively coupled plasma (ICP) test (Table S2, Supporting Information) gives a Cu content of 0.57 wt% and a Ta/Nb ratio of 0.56/0.44. The temperature‐dependent dielectric spectrum was utilized to determine the Curie temperature (*T*_c_) of KTN crystals. As shown in Figure 2d, there is a dielectric permittivity peak at 63.5 °C, indicating there is a ferroelectric–paraelectric phase transition in Cu:KTN. In addition, the differential scanning calorimetry (DSC) data and specific heat (Figure S2, Supporting Information) also give a similar *T*_c_ at 61 °C and *T*_c_ at 58.5 °C, respectively.

Moreover, to evaluate the ferroelectric properties of Cu:KTN, the P–E hysteresis loop was measured at room temperature. The test frequency is 1 Hz. As shown in Figure 2e, the saturation polarization of Cu:KTN crystal is 10.7 µC cm^−2^ and the coercive field is 2.07 kV cm^−1^. In comparison, the saturation polarization of KTN crystal with the same composition recorded is 16.98 µC cm^−2^, and the coercive field is 3.68 kV cm^−1^. We can find that Cu:KTN crystal maintains the ferroelectric properties with slightly reduced saturation polarization and coercive field. This case could be assigned to the reduction of lattice distortion of KTN crystal after Cu^+^ ion doping. In Figure 2f, we add the Cu:KTN in the phase diagram and it is located at the cubic‐tetragonal phase boundary with a low *T*_c_ ≈ 60 °C. According to the previous studies, when the *T*_c_ of KTN crystal is close to room temperature, it is easy to induce the complex ferroelectric domain arrangement and many supercells at micrometer‐scale, thus providing an opportunity to realize the photodetection capacity with high responsivity.^[^ ^19^ ^]^

### 2.3 Ferroelectric‐Order and Supercell Structure in Cu:KTN

**Figure 3:**
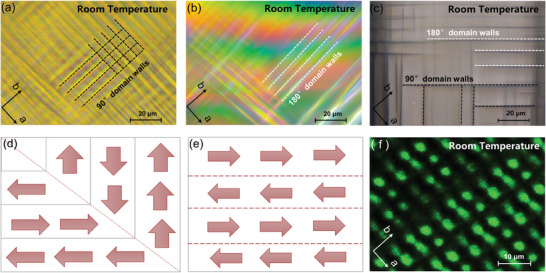
a–c) Domain structure of a Cu:KTN crystal observed under polarizing microscopy. d) Schematic diagram of a 90° domain wall. e) Schematic diagram of a 180° domain wall. f) The second‐harmonic (SH) microscopic pattern of the c‐cut Cu:KTN sample.

As mentioned in the last section, the distribution of ferroelectric domains in KTN with Curie temperature close to room temperature will be very rich. So, we measured the domain structure in Cu:KTN crystal by a polarized light microscope. As shown in **Figure** **3**a–c, the 90° domain walls (black dash line) and the 180° domain walls (white dash line) in Cu:KTN crystal can be observed, which results from the displacement of the B‐site atom in the center of the (BO_6_) octahedron. As a typical perovskite ferroelectric crystal, it is spontaneously polarized along the fourfold axis of the primitive cell with six possible directions. The combination of these different polarization directions allows Cu:KTN crystal to have both 180° and 90° domain walls. The dash lines in Figure 3d,e represent the 90° domain walls and 180° domain walls, respectively. We can see that there are some “head‐to‐head” and “tail‐to‐tail” domain walls, which are favorable to construct conductive channels and improve the photoelectric response.

In addition, the polarization direction of ferroelectric domains can also change the positive and negative nonlinear coefficients of the crystal. The 180° periodic ferroelectric domains can provide reciprocal vectors to compensate for the phase mismatch caused by refractive index dispersion and achieve efficient harmonic output, while the 90° ferroelectric domains cannot^[^ ^52^ ^]^ (Figure S4, Supporting Information). Therefore, domain structures in ferroelectric crystals can be characterized by the second harmonic (SH) image. As displayed in Figure 3f, the green spots originate from the efficient second harmonic generation with the reciprocal vectors provided by the periodic 180° ferroelectric domains, while the dark areas are induced by the 90° domain walls. The size of green spots in the SH image is analyzed to calculate the width of the supercell (Figure S5, Supporting Information), that the narrowest and widest supercell are 1.0 and 2.25 µm, respectively. the cell width with the highest proportion is 1.75 µm. Therefore, we believe that the Cu:KTN crystal still has a supercell structure at micrometer scales.

**Figure 4:**
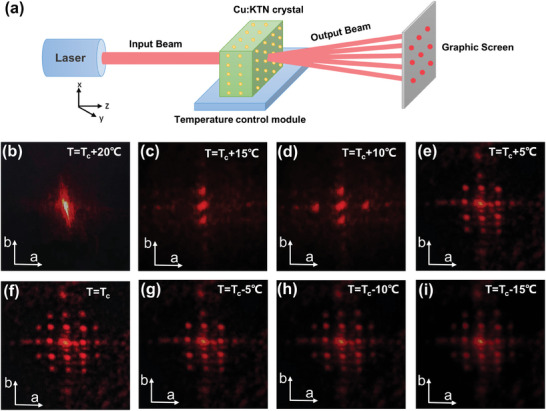
a) Schematic diagram of the Bragg diffraction experiment on Cu:KTN crystal. Superlattice diffraction pattern of Cu:KTN crystal at b) *T*_c_ + 20 °C, c) *T*_c_ + 15 °C, d) *T*_c_ + 10 °C, e) *T*_c_ + 5 °C, f) *T*_c_, g) *T*_c_ − 5 °C, h) *T*_c_ − 10 °C, and i) *T*_c_ − 15 °C.

Moreover, to explore the existence of Cu:KTN crystal superlattice structure in the crystal and the effect of this structure on the optical diffraction effect, we carried out the Bragg diffraction experiments at various temperatures. The experimental device is plotted in **Figure** **4**a, where the wavelength of the incident He–Ne laser is 633 nm. The temperature of Cu:KTN crystal is controlled by a thermal module with a temperature range of *T*_c_ − 15 °C ≈ *T*_c_ + 20 °C. An optical screen records the far‐field diffraction spot. We find that the diffraction effect of the Cu:KTN crystal varies with the crystal temperature, and the diffraction phenomenon of the superlattice below the Curie temperature is similar to the optical diffraction phenomenon of X‐ray diffraction, which indicates that there should be 3D supercell structure in Cu:KTN crystal at micrometer scales.

When *T* > *T*_c_, Cu:KTN crystal belongs to the paraelectric phase and there are no supercell structures in the crystal. At this time, only a bright spot can be observed on the screen (Figure 4b) and there is no diffraction pattern when the laser irradiates the crystal. As the temperature gradually decreases, the supercell structures in Cu:KTN crystal begin to form and the diffraction phenomenon begins to appear. The typical diffraction pattern is shown in Figure 4c–e. When the temperature decreases to *T*_c_, the supercell structure is very complex and the diffraction pattern on the optical screen also becomes complicated (Figure 4f). As the temperature continues to drop, the crystal completely transforms into the ferroelectric phase. Due to the influence of the ferroelectric domain scattering, the transmission efficiency of the He–Ne laser is seriously reduced, and the brightness begins to decline and becomes fuzzy (Figure 4g–i). Therefore, this temperature‐dependent Bragg diffraction demonstrated that there are rich ferroelectric supercells in as‐grown Cu:KTN crystal. In addition, we performed the Bragg diffraction experiments under *T* = *T*_c_ with other wavelengths at 594, 543, and 456 nm, respectively (Figure S6, Supporting Information), which are consistent with diffraction at 633 nm.

The second harmonic pattern and Bragg diffraction both prove the existence of 3D supercells composed of “head‐to‐head” and “tail‐to‐tail” ferroelectric domains in Cu:KTN crystal. In previous reports, such 3D supercells in pure KTN crystal^[^ ^52^ ^]^ are sufficient to promote highly‐conductive channels and improve the self‐powered photocurrent (Figure S7, Supporting Information). Therefore, the bifunctional strategy is successfully achieved in Cu:KTN, that the absorption edge redshifts to 1100 nm by Cu‐doping and 3D supercell ferroelectric‐order induced by tailored ferroelectric polarization. Therefore, Cu:KTN crystal is expected to achieve extremely high self‐powered responsivity in a wide spectral range.

### 2.4 Performances of Self‐Powered Cu:KTN Photodetector

**Figure 5:**
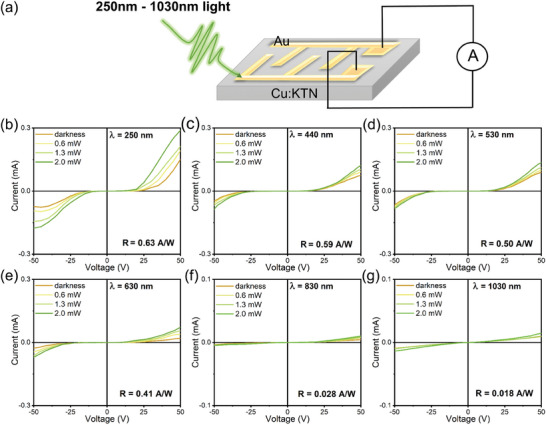
a) Schematic diagram of Cu:KTN crystal photodetector. The current–voltage (*I*–*V*) relationship of the Cu:KTN based photodetector at b) 250, c) 440, d) 530, e) 630, f) 830, and g) 1030 nm.

Then, we fabricated the Cu:KTN photodetector and measured their performance at room temperature. The experimental setup is shown in **Figure** **5**a. Light‐emitting diodes of different wavelengths are used as incident light sources. We use the responsivity *R* = *I*_ph_/*P* to measure the photoelectric detection capability of the device, where *P* is the average power of the incident light, *I*_ph_ is the net photocurrent, and *I*_ph_ = *I*_illumination_ − *I*_darkness_. The higher the responsivity *R*, the more sensitive the device is to light.

We measured the photoelectric response of the Cu:KTN crystal under a 250 nm light source. Due to the presence of ferroelectric domains in the tetragonal Cu:KTN phase, the carrier transport is affected by additional mechanisms besides Ohm's law, and the crystal exhibits a nonlinear *I*–*V* curve. When the incident light power is 2 mW, the maximum photocurrent of the Cu:KTN photodetector reaches 0.29 mA with the bias voltage of 50 V. At this time, the corresponding responsivity is 0.63 A W^−1^ (Figure 5b). In addition, Cu:KTN detector can also achieve a self‐powered response in the ultraviolet band. When the incident light power is 2 mW, the self‐powered photocurrent is 32.6 nA, and the corresponding responsivity is 5.23 mA W^−1^(Figure S8, Supporting Information). This value is fifty times higher than that of pure KTN crystal (0.11 mA W^−1^).

**Figure 6:**
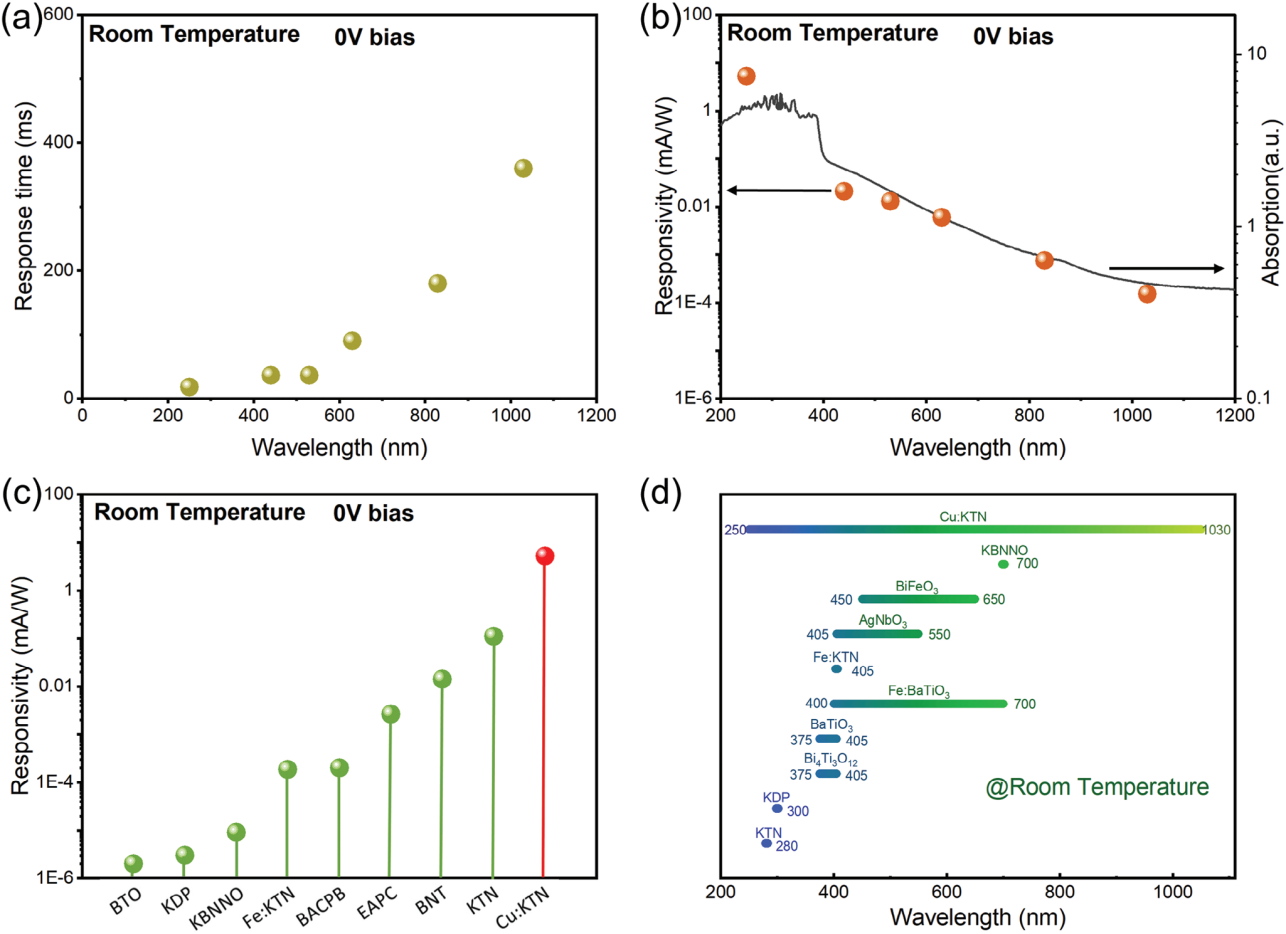
a) Wavelength‐dependent response time of Cu:KTN detector at zero bias voltage. b) Wavelength‐dependent responsivity of Cu:KTN detector at zero bias voltage. The absorption spectrum was plotted in log form. c) Comparison of self‐powered responsivity of BaTiO_3_ (BTO),^[^ ^55^ ^]^ KH_2_PO_4_ (KDP),^[^ ^31^ ^]^ [KNbO_3_]_1−_ *_x_* [BaNi_1/2_Nb_1/2_O_3−&_]*_x_* (KBNNO),^[^ ^33^ ^]^ Fe:KTa_0.41_Nb_0.59_O_3_ (Fe:KTN59),^[^ ^50^ ^]^ BA_2_CsPb_2_Br_7_ (BACPB),^[^ ^35^ ^]^ EA_4_Pb_3_Cl_10_ (EAPC),^[^ ^36^ ^]^ Bi_0.5_Na_0.5_TiO_3_ (BNT),^[^ ^37^ ^]^ KTa_0.59_Nb_0.41_O_3_ (KTN41),^[^ ^19^ ^]^ and Cu:KTa_0.56_Nb_0.44_O_3_ (Cu:KTN44) for ultraviolet light. d) Comparison of photoelectric response range of self‐powered detectors, including KTa_0.59_Nb_0.41_O_3_ (KTN41),^[^ ^19^ ^]^ KH_2_PO_4_ (KDP),^[^ ^31^ ^]^ Bi_4_Ti_3_O_12_,^[^ ^12^ ^]^ BaTiO_3_,^[^ ^55^ ^]^ Fe:BaTiO_3_,^[^ ^56^ ^]^ Fe:KTa_0.41_Nb_0.59_O_3_ (Fe:KTN59),^[^ ^50^ ^]^ AgNbO_3_,^[^ ^34^ ^]^ BiFeO_3_,^[^ ^30^ ^]^ [KNbO_3_]_1−_ *_x_* [BaNi_1/2_Nb_1/2_O_3−&_]*_x_* (KBNNO),^[^ ^33^ ^]^ and Cu:KTa_0.56_Nb_0.44_O_3_ (Cu:KTN44).

Moreover, to explore the extended photo‐responsive capacity in Cu:KTN crystals, we tested the *I–V* curves at different incident wavelengths (Figure 5c–g). It is optical active at visible and near‐infrared range (440–1030 nm). When the bias voltage is 50 V and the incident light power is 2 mW, the responsivity of Cu:KTN detector are 0.59, 0.50, 0.41, 0.028, and 0.018 A W^−1^, at 440, 530, 630, 830, and 1030 nm, respectively. The current–time (*I–T*) curves show that the self‐powered responsivity of Cu:KTN detector is 0.021 mA W^−1^, 0.013 mA W^−1^, 0.006 mA W^−1^, 0.750 µA W^−1^, and 0.145 µA W^−1^, respectively (Figure S8, Supporting Information). It is preliminarily inferred that the variation of the responsivity of Cu:KTN photodetector may be related to its absorption capacity to various light sources. The response time of Cu:KTN crystal in these six wavelengths is 16, 32, 32, 80, 160, and 320 ms, respectively (**Figures** **6**a and S9, Supporting Information). In addition, to explore the reasons for the decreased responsivity of Cu:KTN crystal at the near‐infrared region, we draw the responsivity intensity at different wavelengths and the absorption spectrum of Cu:KTN in Figure 6b. It is observed that the scatter points are evenly distributed on both sides of the absorption spectrum, and the variation trend of responsivity is well consistent with the absorption spectrum. Therefore, we conclude that the decreased responsivity of Cu:KTN detectors at long wavelengths could be attributed to the reduced absorption of different light sources.

Ferroelectric Cu:KTN is a non‐centrosymmetric crystal, which has frequency‐doubling capacity. Accordingly, its responsive range can be further extended by nonlinear optical up‐conversion.^[^ ^52^ ^]^ For example, limited by the weak absorption capacity, Cu:KTN is inactive for a 1240 nm laser. However, through a nonlinear frequency‐doubling process, the 1240 nm laser can be converted to a 620 nm red laser, which leads to a strong detection signal. As shown in Figure S10 (Supporting Information), we investigated the photoelectric response of Cu:KTN detector using two light sources, 1240 nm light‐emitting diode (LED) and 1240 nm femtosecond pulsed laser. For 1240 nm LED irradiation, the photocurrent of Cu:KTN detector is zero. In comparison, for a femtosecond pulsed laser at 1240 nm, Cu:KTN detector becomes active and the self‐powered responsivity is 0.023 µA W^−1^ (Figure S10, Supporting Information). In theory, we can use this nonlinear frequency‐doubling effect to extend the responsive range of Cu:KTN crystal to 2000 nm, covering the optical communication bands.

**Figure 7:**
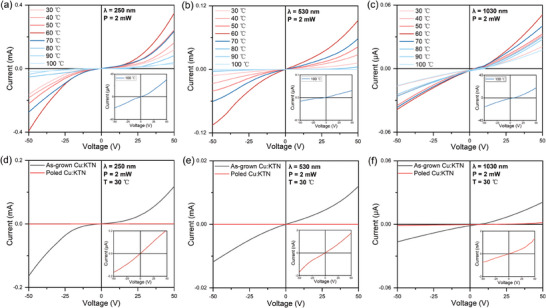
The temperature‐dependent current–voltage (*I–V*) curves of Cu:KTN crystal at a) 250, b) 530, and c) 1030 nm. The insets are the enlarged *I–V* curves of Cu:KTN crystal at 100 °C. The *I–V* curves of the as‐grown and poled Cu:KTN crystal at d) 250, e) 530, and f) 1030 nm. The insets are the enlarged *I–V* curves of the poled Cu:KTN crystal.

Furthermore, in order to further explore the relationship between photoelectric performance and ferroelectricity of Cu:KTN crystal, we tested the *I–V* curves from 30 to 100 °C, above the Curie temperature (**Figure** **7**a–c; Figure S11 and Table S3, Supporting Information). It is found that the photocurrent of the crystal shows the same trend with temperature at different wavelengths. At *T* < *T*_c_, the photocurrent gradually increases with the temperature rising. When the temperature rises to *T*_c_, the photocurrent reaches maximum, which could be assigned to the ferroelectric domain arrangements and the emergence of micrometer‐scale supercells. Since the temperature continues to rise, the Cu:KTN crystal gradually changes from ferroelectric to paraelectric phase, and the ferroelectric domain disappear. As a result, the photocurrent decreases with the increasing temperatures. When the temperature rises to 100 °C, the *I–V* curve shows a near‐linear behavior following a simple Ohm's law, which is consistent with our previous report on cubic KTN.^[^ ^19^ ^]^ Moreover, it is obvious that this photocurrent change could be well explained by the change of ferroelectric polarization at different temperatures in Figure 4, thus addressing that the excellent of Cu:KTN detector is dominated by the ferroelectric polarization, not semiconductor. It is worth pointing out that there are still non‐linear photocurrent persisted in the paraelectric phase up to the temperature around *T* = *T*_c_ + 50 °C, which could be assigned to the possible polar nanoregions (PNRs) in Cu:KTN.^[^ ^53^, ^54^ ^]^ These PNRs may induce complex domain arrangements in the ferroelectric phase in which many supercells appear in the micrometer range.

Meanwhile, we tested the photocurrent of Cu:KTN crystal before and after the poling treatment (Figure 7d–f and Figure S12, Supporting Information). The photocurrent of the poled Cu:KTN crystals decreases significantly at the three wavelengths. At 50 V bias, the comparison of photocurrent between the single‐domain Cu:KTN crystal and the as‐grown Cu:KTN crystal shows a higher photocurrent more than three orders of magnitude. This could be assigned to the disappeared ferroelectric supercells in the poled Cu:KTN crystal. This could be assigned to the disappeared ferroelectric supercells in the poled Cu:KTN crystal. Therefore, this further demonstrates that the photoelectric response of Cu:KTN self‐powered detector is dependent on the exotic ferroelectric‐orders with “head‐to‐head” and “tail to‐tail” ferroelectric domains.

Finally, we compare the photoelectric detection performances of several self‐powered detectors based on ferroelectric materials (Figure 6c). We can see that our fabricated Cu:KTN detector exhibits the highest responsivity, which is fifty times higher than undoped KTN crystal^[^ ^19^ ^]^ (*R* = 0.11 mA W^−1^ at 280 nm) and four orders of magnitude higher than the orthorhombic Fe‐doped KTN crystal (*R* = 1.85 × 10^−4^ mA W^−1^ at 405 nm).^[^ ^41^, ^50^ ^]^ In addition, the detection performance of Cu:KTN is also better than other ferroelectric film and hybrid ferroelectrics, such as BNT (*R* = 0.014 mA W^−1^ at 365 nm),^[^ ^37^ ^]^ EAPC (*R* = 2.62 × 10^−3^ mA W^−1^ at 206 nm),^[^ ^36^ ^]^ BACPB (*R* = 2 × 10^−4^ mA W^−1^ at 405 nm),^[^ ^35^ ^]^ and so on. Figure 6d depicts a comparison of the photoelectric response ranges of self‐powered ferroelectric detectors. In many oxide ferroelectrics with large band gaps, the response interval is limited in the ultraviolet region, comprising KTN,^[^ ^19^ ^]^ KDP,^[^ ^31^ ^]^ BaTiO_3_,^[^ ^55^ ^]^ and Bi_4_Ti_3_O_12_.^[^ ^12^ ^]^ The longest response wavelength is only 405 nm. In 2019, AgNbO_3_ ceramics with a band gap of 2.75 eV were prepared with an extended response range to 550 nm.^[^ ^34^ ^]^ In 2021, Li et al prepared a BiFeO_3_ thin film with a band gap of 2.05 eV and extended the response range to 650 nm by defect engineering.^[^ ^30^ ^]^ In 2020, Fe‐doped BaTiO_3_ was reported to exhibit an extended response range to 700 nm by introducing donor and accepter energy levels.^[^ ^56^ ^]^ However, all these self‐powered detectors are still limited in the visible region. It is observed that the response range of Cu:KTN covers the entire visible region and extends to the near‐infrared region for the first time (250–1030 nm), which is the widest among all ferroelectric‐based self‐powered detectors. Combining the ultra‐high responsivity and broadband responsive range, Cu:KTN crystal would be a potential self‐powered ferroelectric photodetector for those miniaturized on‐chip applications.

## 3 Conclusion

We proposed a bifunctional design strategy in bulk ferroelectric crystals to fabricate a high‐performance self‐powered photodetector. Using Cu:KTN as an example, the redshift absorption spectra and micrometer‐scale supercell were realized concurrently. A giant self‐powered responsivity of 5.23 mA W^−1^ was achieved in Cu:KTN detector and the responsive range was also extended from 250 to 1030 nm. To the best of our knowledge, this represents the widest response range among all ferroelectric‐based self‐powered detectors. This ion‐doping design strategy should be appliable for many ferroelectric crystals, such as BaTiO_3_, LiNbO_3_, and SBN. Besides Cu‐doping, near‐infrared and min‐infrared responses could be expected by selecting suitable doping ions, such as Cr^3+^, Ti^4+^, and Fe^2+^. This work not only provided a rational design strategy for improving the performance of ferroelectric detectors, but also facilitated the development of other ferroelectric‐based optoelectronic applications, such as ferroelectric photovoltaic, ferroelectric memory, and nonlinear holography.

## 4 Experimental Section

### Cu:KTN Crystal Growth

The Cu:KTN crystal used in this experiment was obtained by the TSSG method. Pure KTN crystals oriented along the *c*‐axis were placed as seed crystals 10 mm from the center of the platinum crucible. High‐purity K_2_CO_3_, Nb_2_O_5,_ and Ta_2_O_5_ powders were mixed in a certain proportion according to the phase diagram as raw materials used for growth. During the growth process, CuO with a 5% mass fraction and K_2_CO_3_ with a 5% mass fraction were added as dopants and co‐solvents, respectively. During crystal growth, the lifting speed was set to 0.4 mmh^−1^ and the rotation speed was set to 8 rpm. After the growth was completed, the crystal was processed into a regular cuboid with a size of 4 mm × 8 mm × 2 mm in the direction of [001] and polished.

### Crystal Characterization

The XRD pattern of Cu:KTN crystal was obtained by a high‐resolution X‐ray diffractometer (Smartlab 3KW) and a Cu‐Ka target (*λ* = 1.5406 Å) with an accuracy of 1° min^−1^. The test range was 10–80°. The XPS was performed with an aluminum target and a monochromatic Ka source, where the integral area of each peak represented the content of each element in the crystal. The absorption spectra of Cu:KTN and KTN crystal were obtained by UV–vis spectrophotometer (UV‐2600i) with a step length of 0.5 nm and test range of 200–1400 nm. The hysteresis loop of Cu:KTN crystal was obtained by a ferroelectric test system (RT‐Precision LC, Radiant Technology) at the test frequency of 1 Hz and the test temperature was room temperature. The differential scanning thermal analysis (DSC) map was obtained by the differential scanning calorimeter (Mettler DSC3). The test temperature range was from room temperature to 200 °C, the gas atmosphere was N_2_, the test mode was rising and cooling curve, and the speed was 10 °C min^−1^. The Specific heat test was obtained by the differential scanning calorimeter (Mettler DSC3). The test temperature range was from room temperature to 200 °C, the gas atmosphere was N_2_, and the speed was 10 °C min^−1^. The second‐harmonic light of the Cu:KTN crystal was obtained by using a 1030 nm laser incident with a repetition rate of 2 MHz and a pulse width of 250 fs.

### Theoretical Calculations

The density functional theory calculations were performed by CASTEP package. The tetragonal KTN (P4mm space group) were used as primitive cells. The crystal cell parameters of KTN and the position of the atoms in the crystal cell were optimized. The optimized norm‐conserved pseudopotentials and the Monkhorst–Pack k‐point grid (6 × 6 × 6) were used in the Brillouin region, and a kinetic cutoff of 500 eV was selected. The optimized crystal structure of KTa_0.6_Nb_0.4_O_3_ (*a* = 3.998 Å, *c* = 4.097 Å) agreed well with the experimental value (*a* = 3.996 Å, *c* = 4.063 Å). A 3 × 3 × 3 KTN supercell was constructed. Two possible substitution sites of Cu^+^ were considered at the A‐site and the B‐site, respectively.

### Photoelectric Detection Experiment

A [001]‐orientated Cu:KTN sample was utilized with 8 mm × 4 mm × 2 mm size. A magnetron sputtering instrument (KT‐1650PVD) and a mask plate were used to sputter an Au interdigital electrode on the crystal surface. The sputtering current was 200 mA and the sputtering time was 90 s. The electrode thickness was 10 nm and the finger electrode width was 0.1 mm. The light source was commercialized LED, and the incident light wavelength was 250, 440, 530, 630, 830, and 1030 nm. The photocurrent was collected by the Keithley‐2450 digital source meter, which obtains the signal by contacting the finger electrode on the crystal surface with a conductive probe. Two different Cu:KTN samples with different doping concentrations (0.57 and 1.36 wt%) was tested (Figure S13, Supporting Information). The influence of electrode was also evaluated by different Cu:KTN detectors with Au‐, Ag‐, and Cu‐electrode (Figure S14, Supporting Information).

## Conflict of Interest

The authors declare no conflict of interest.

## Author Contributions

F.L. and H.H.Y. conceived and supervised this project. Y.Q.W. and Y.B.W. performed optical experiments and wrote the manuscript. X.P.W. grew the KTN and Cu:KTN crystals. H.J.Z. and Y.C.W. provided helpful suggestions for the design of ferroelectric domains in Cu:KTN crystal. All authors contributed to the discussion and preparation of the manuscript.

## Supporting information



Supporting Information

## Data Availability

The data that support the findings of this study are available in the supplementary material of this article.
