# Supplementary material for: Bifunctional Design of Ferroelectric‐Order and Band‐Engineering in Cu:KTN Crystal for Extended Self‐Powered Photoelectric Response
Source: Adv Sci (Weinh). 2024 Dec 17;12(6):2412877. doi: 10.1002/advs.202412877 (PMC11809387; doi:10.1002/advs.202412877)
Supplement: Supplementary file 1 — Supporting Information [file ADVS-12-2412877-s001.docx]

**Supporting Information**

**Bifunctional design of ferroelectric-order and band-engineering in Cu:KTN crystal for extended self-powered photoelectric response**

Yaqian Wang^1^, Yabo Wu^1,2^, Fei Liang^1*^, Xuping Wang^3*^, Haohai Yu^1*^, Huaijin Zhang^1^, Yicheng Wu^1^

^1^ State Key Laboratory of Crystal Materials and Institute of Crystal Materials, Shandong University, Jinan 250100, China

^2^ Xinjiang Technical Institute of Physics and Chemistry, Chinese Academy of Sciences, Urumqi 830011, China

^3^ Advanced Materials Institute, Qilu University of Technology (Shandong Academy of Sciences), Jinan 250014, China

Corresponding author*

Email: [liangfei@sdu.edu.cn](mailto:liangfei@sdu.edu.cn) ( Fei Liang)

Email: [wangxp@sdas.org](mailto:wangxp@sdas.org) (Xuping Wang)

Email: [haohaiyu@sdu.edu.cn](mailto:haohaiyu@sdu.edu.cn) (Haohai Yu)

**1. Additional Figures and Tables**

**1.1 Characterization of Cu ion valence state in Cu:KTN crystal**


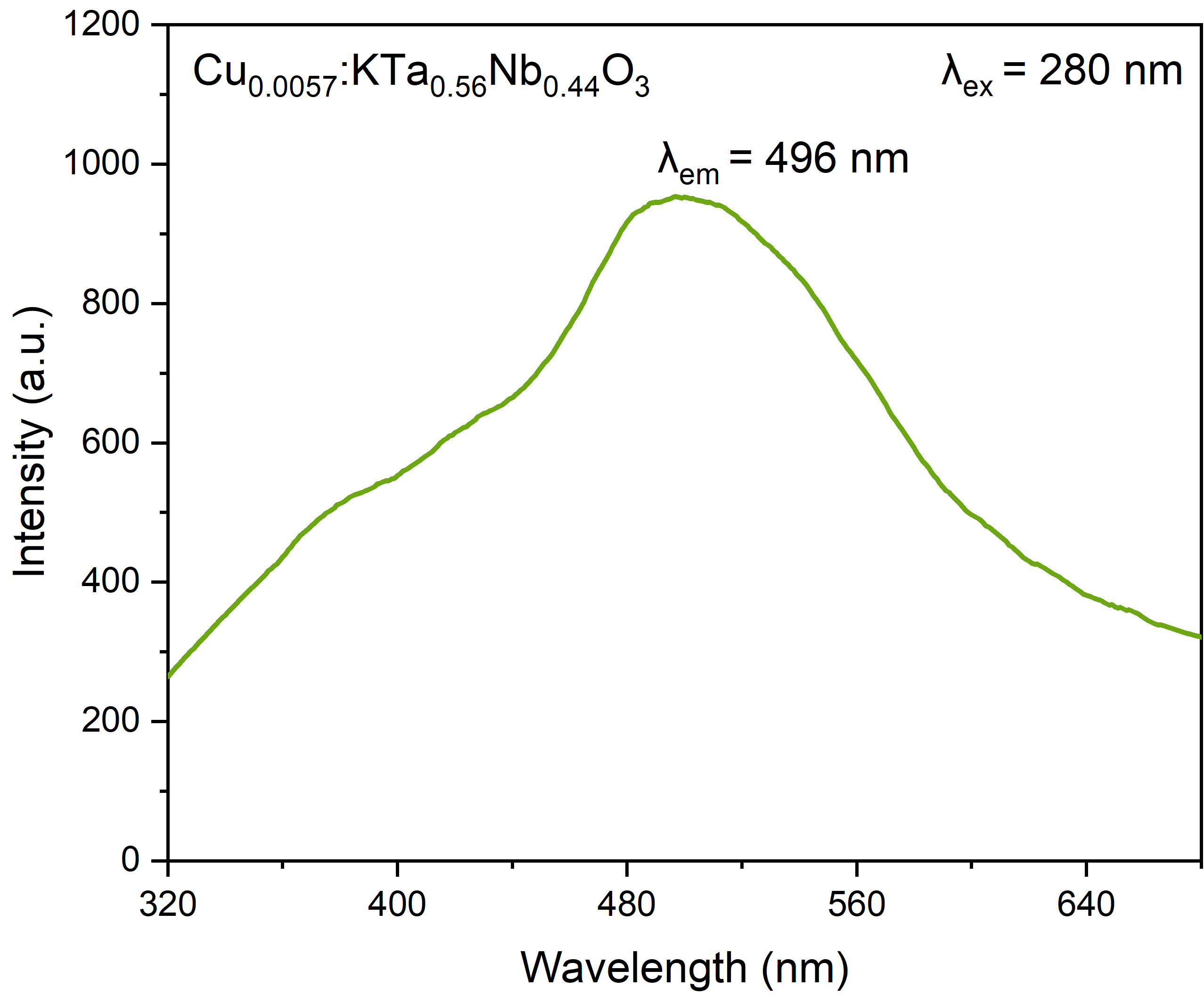


**Figure S1.** The fluorescent emission spectra of Cu:KTN crystal.

To investigate the valence state of Cu ions in Cu:KTN crystal, we measured its fluorescence spectrum under room temperature. The excitation wavelength is 280 nm. As shown in Figure S1, a broad emission band appears in the range of 350-700 nm and the fluorescent peak is located at 496 nm. This peak is close to the situation of Cu^+^-doped borosilicate glass and NaCl crystal, that located at 480 nm and 503 nm, respectively^[1-3]^.

In addition, we measured the fluorescent spectra at the excitation wavelength of 286 nm and 347 nm. There is no fluorescent emission peak near 600 nm, thereby precluding the possibility of Cu^2+^ in Cu:KTN crystal. So we can reasonably conclude that Cu^+^ is the dominated type in Cu:KTN crystal.

**1.2 Curie temperature characterization of crystals**


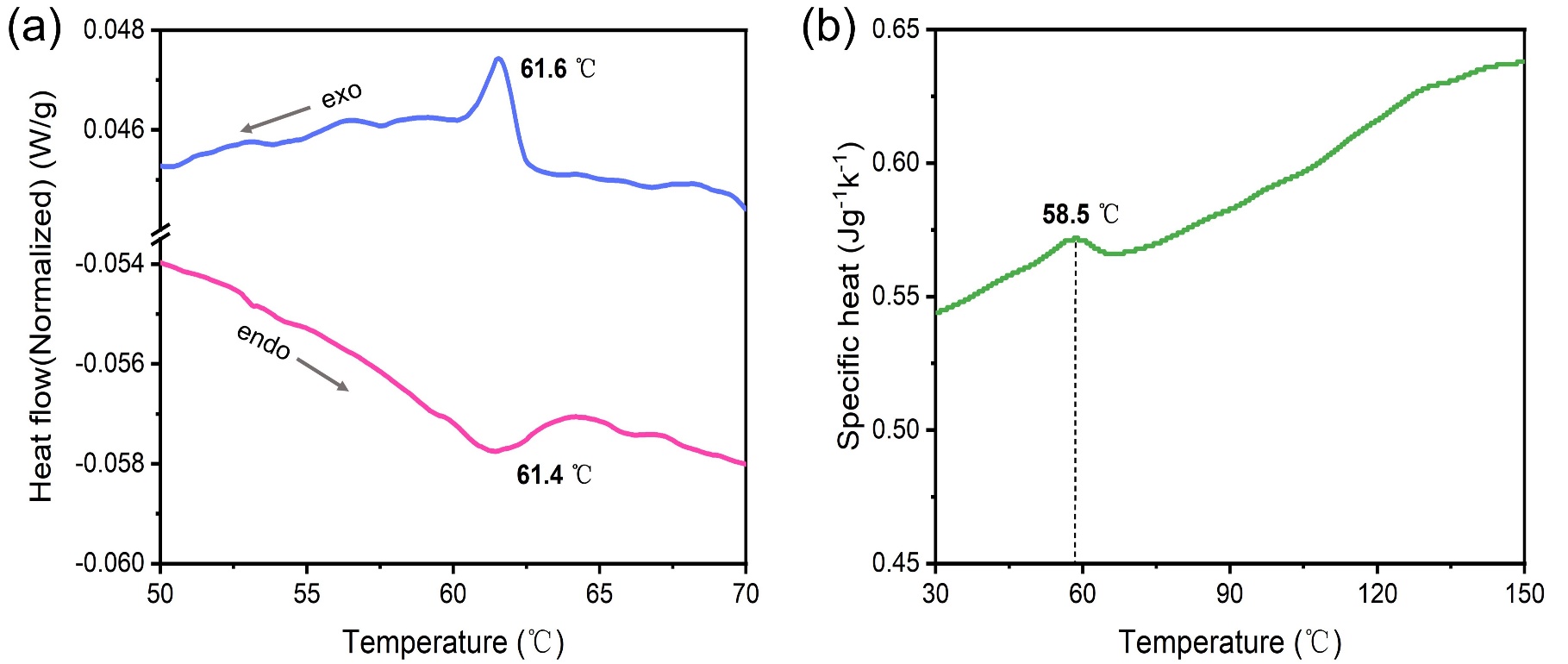


**Figure S2**. a) Differential scanning thermal analysis (DSC) map of Cu:KTN crystal. b) Specific heat of Cu:KTN crystal.

We tested the relationship between the specific heat and temperature of the crystal by differential scanning calorimetry, and drew the DSC curve of the crystal, as shown in Figure S2. The red line and blue line in the DSC spectrum represent the DSC effect curve of Cu:KTN crystal from 10 ℃ to 70 ℃ and from 70 ℃ to 10 ℃, respectively. We find that the phase transition heat absorption peak and heat release peak of the crystal are around 60 ℃, and the specific heat value also changes at 58.5 ℃.

**1.3 The Raman spectrum of Cu:KTN crystals**


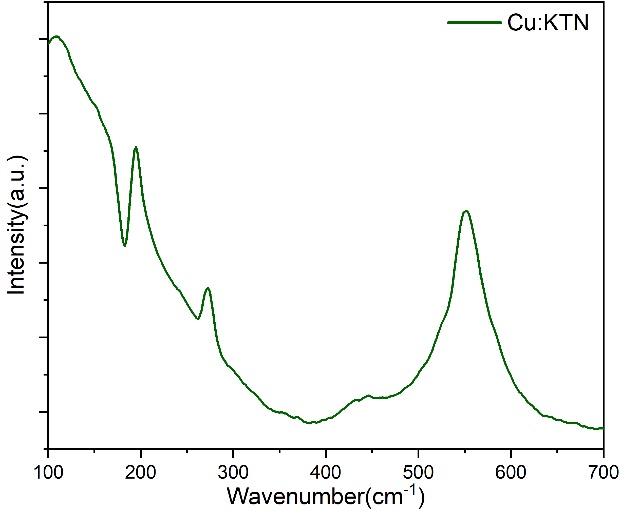


**Figure S3.** The Raman spectrum of Cu:KTN crystals.

It is observed that the emergence of asymmetric Fano resonance at 198 cm^-1^. This Fano resonance originates from the interference of a localized discrete state coupled to the continuum states. In tetragonal Cu:KTN, it is noted that the difference between d(Nb-O) and d(Ta-O) is compatible with the Fano resonance mechanism, that is, the broad Nb-O vibrations provide the continuous background and the Ta-O vibrations provide a sharp discrete state^[4]^.

**1.4 Ferroelectric-order and super-crystal structure in Cu:KTN**


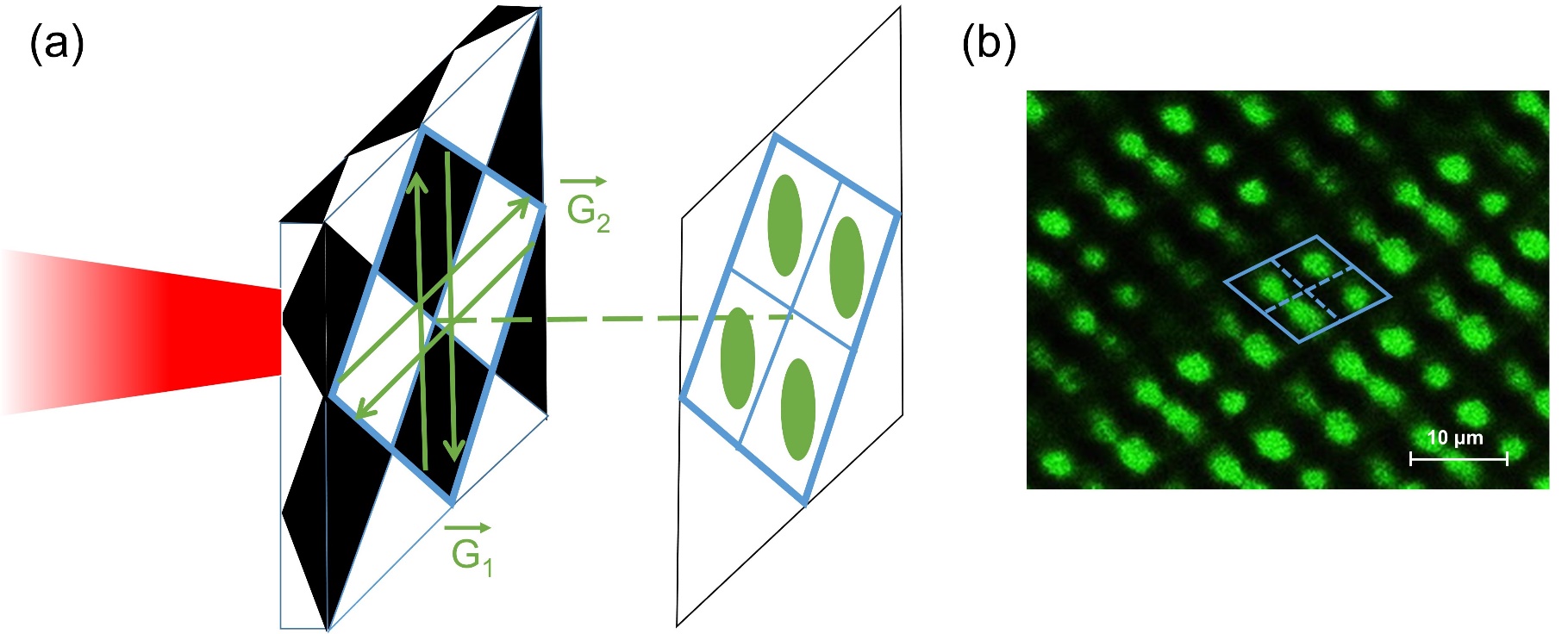


**Figure S4.** a) Schematic diagram of second harmonic imaging of Cu:KTN crystals (black and white regions represent periodic 180° ferroelectric domain regions). b). Cu:KTN crystal second harmonic imaging pattern.

The ferroelectric domain structure in KTN crystals can be characterized by second harmonics. As shown in Figure S4a, periodic 180° ferroelectric domains in the black and white regions can provide reciprocal vectors along both directions to compensate for the phase mismatch due to refractive index dispersion, which results in a second harmonic output in both horizontal and vertical directions (green spots in Figure S4b). In contrast, the boundary of the orthogonal ferroelectric domain region along the 45° direction is the 90° domain wall, and it can not provide a significant reciprocal lattice to meet the requirements for generating second harmonics, resulting in the dark spots in Figure S4b.

**1.5 Size distribution statistics of supercells in Cu:KTN**


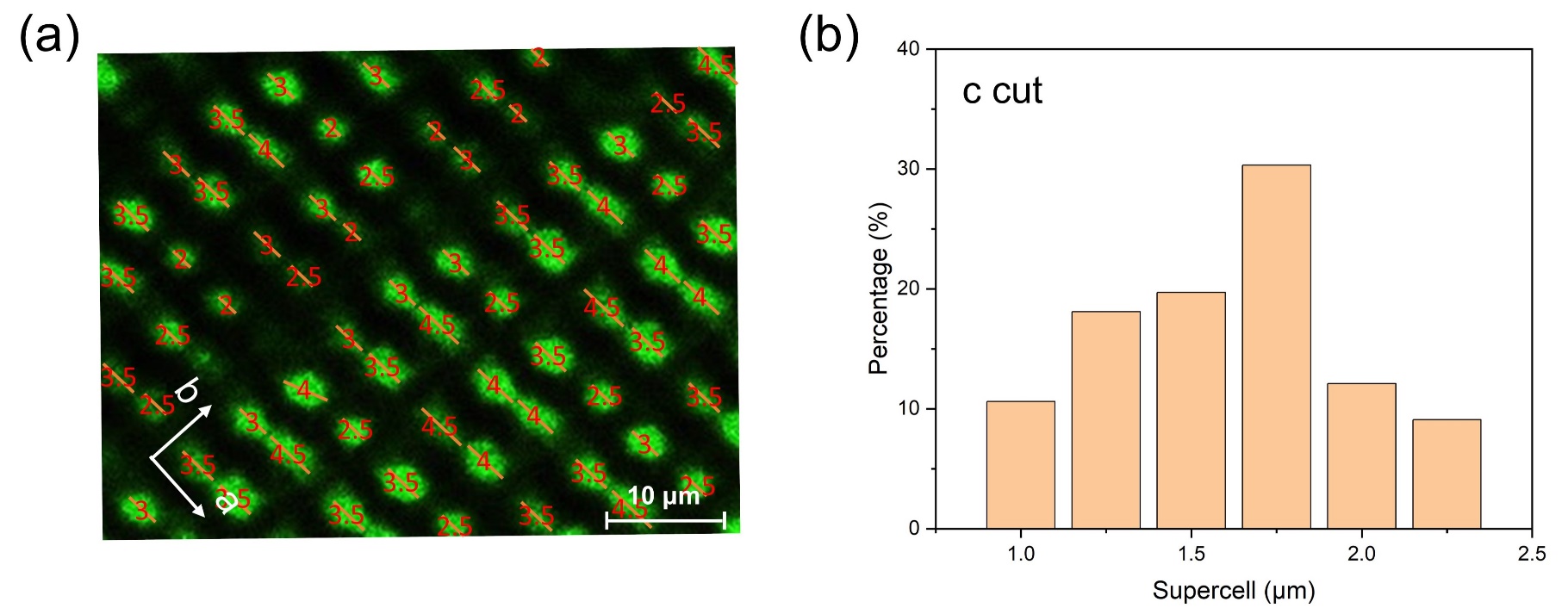


**Figure S5.** a) Supercell size statistics in the SH pattern of Cu:KTN crystal. b) Statistical distributions of supercells in c-cut Cu:KTN crystal

The green spots in the SH image represent a pair of inverted 180° ferroelectric domains with a width equal to twice the inverse-period 180° ferroelectric domain width, so the size of the supercell can be calculated from the size of the green spots. First, we set a scale of 10 μm (bottom right white line) to define the overall size of the SH graph. Then, we select the longest width of each SH point, draw orange lines as the spot size, and then calculate the size of the supercell. We draw 66 orange lines in Figure S5a. Based on origin software measurement, the supercell width of c-cut crystals is statistically measured, as shown in Figure S5b. The narrowest and widest supercells are 1.0 μm and 2.25 μm, respectively. The most distributed cell size is 1.75 μm.

**1.6 Superlattice diffraction pattern of Cu:KTN crystal at Tc**


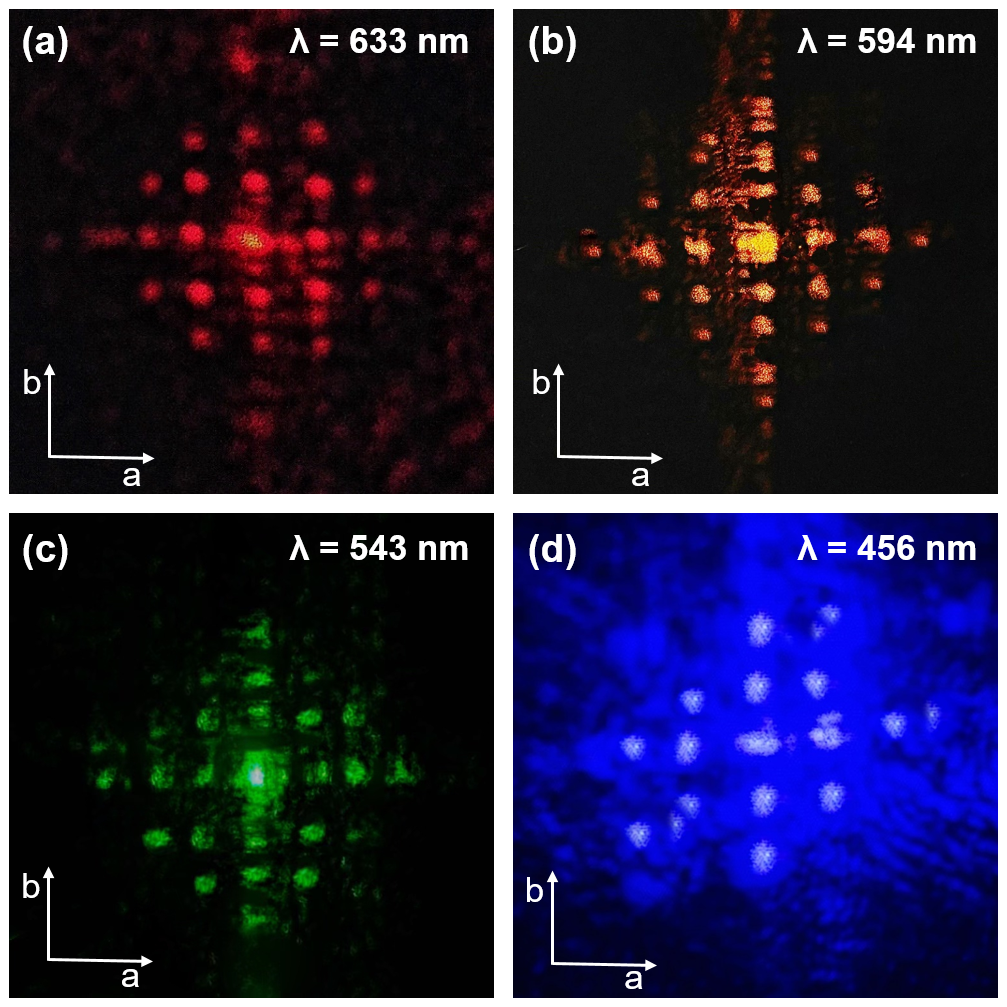


**Figure S6**. Superlattice diffraction pattern of Cu:KTN crystal at a) 633 nm, b) 594 nm, c) 543 nm, d) 456 nm.

To explore the existence of Cu:KTN crystal superlattice structure in the crystal and the effect of this structure on the optical diffraction effect, we carried out the Bragg diffraction experiments at T_c_, with the incident laser wavelengths were 633 nm, 594 nm, 543 nm, and 456 nm respectively. We find that Cu:KTN crystals exhibit the obvious Bragg diffraction patterns at different wavelengths. According to Bragg diffraction formula, the first-order Bragg diffraction angle of Cu:KTN crystals satisfies the following conditions, 2dsin*θ*=λ. By measuring the diffraction angle, we calculate that the cell constant *d* is about 2.0 μm, which is consistent with the supercell size reported in the previous paper [Light: Science & Applications 9, 193 (2020)].

**1.7 Structural model of the ferroelectric domain in the Cu:KTN**


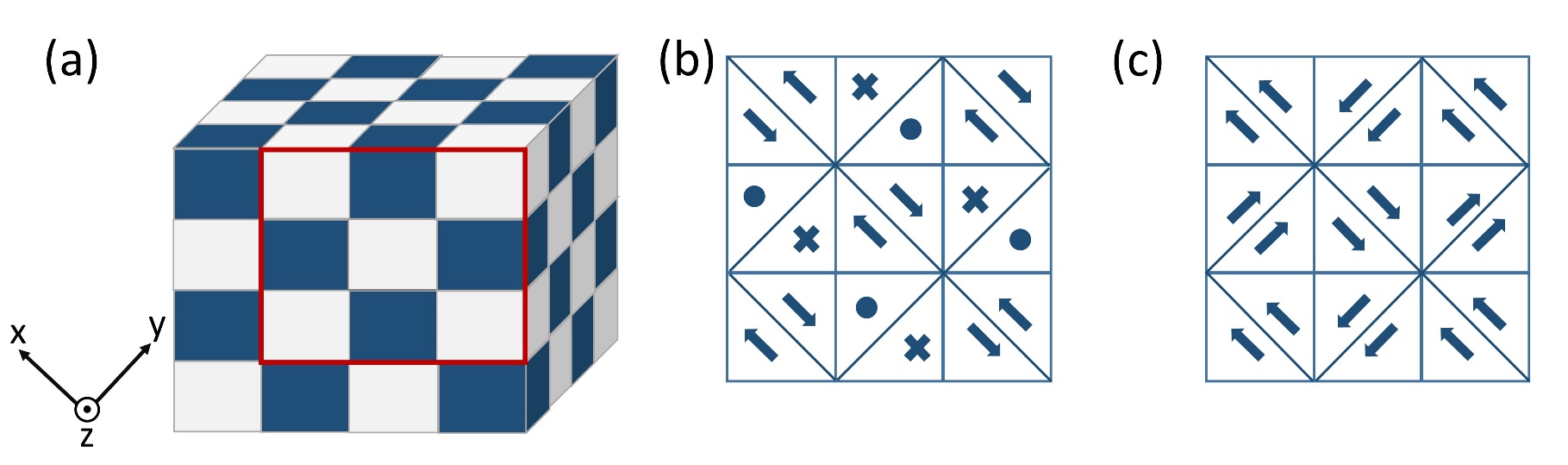


**Figure S7**. a) The diagram of the ferroelectric domain in the three-dimensional supercells of Cu:KTN. b) Structural model of the ferroelectric domain (out-of-plane) in the Cu:KTN x–y plane. c) Structural model of the ferroelectric domain (in-plane) in the Cu:KTN x–y plane.

In the three-dimensional space of the Cu:KTN crystal, the 180° domain wall and the 90° domain wall are combined into a new periodic structure, and they are embedded with each other to form a supercell, which is periodically arranged in a crystal with a Rubik's cube pattern (Figure S7a). At the same time, the ferroelectric domain in the supercell can form a new three-dimensional structure. As shown in Figure S7b and c, there are both up and down ferroelectric polarizations in the crystal along the x, y, and z directions. These ferroelectric polarizations combine with each other to form a "head-to-head" and "tail-to-tail" conductive domain wall, which causes the fluctuation of electrostatic potential in the crystal, accelerates the separation of charge carriers, and achieves high photoelectric response.

**1.8 The current-time relationship of the Cu:KTN**


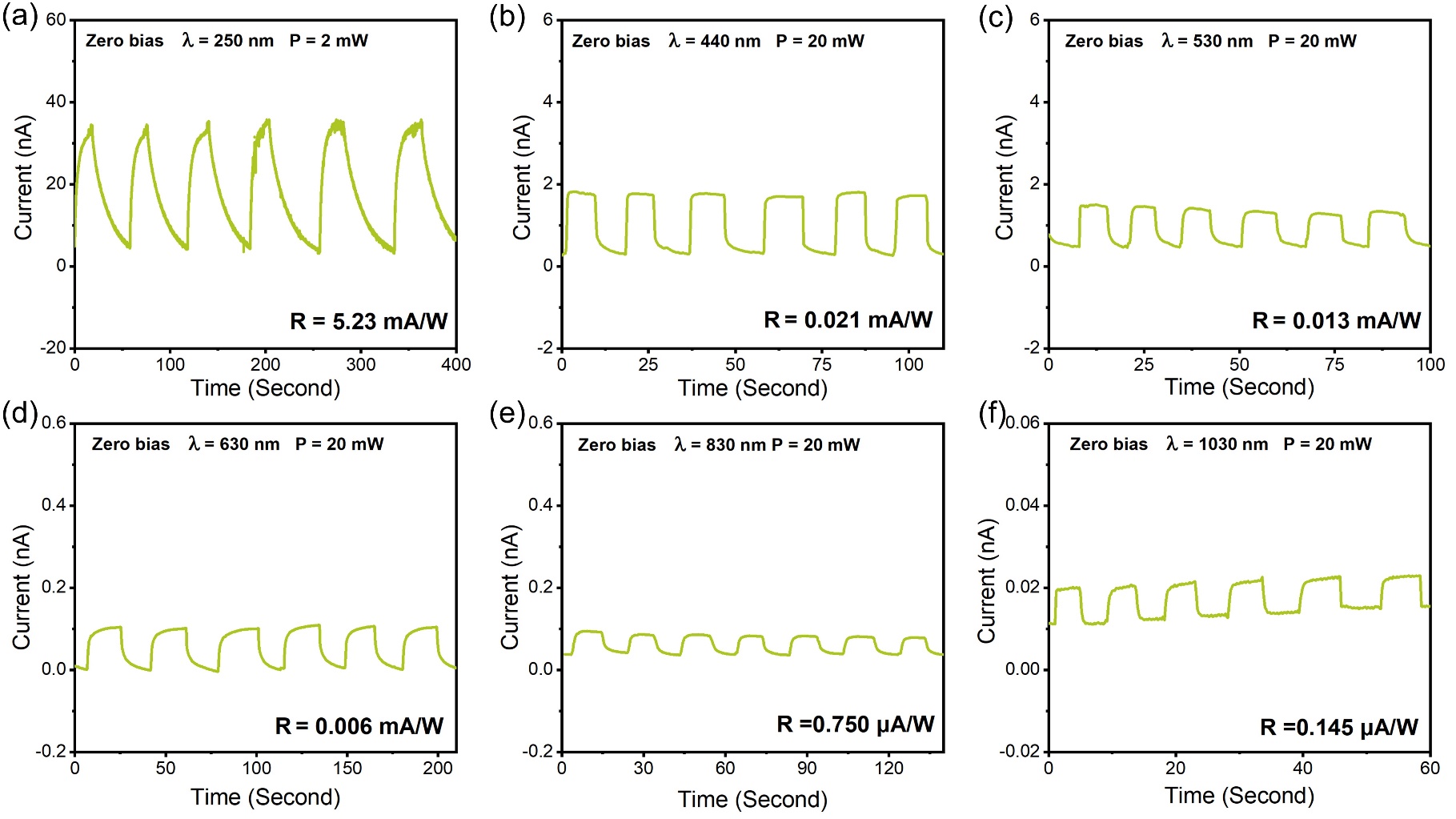


**Figure S8**. The current-time (I-T) relationship of the Cu:KTN based photodetector at a) 250 nm, b) 440 nm, c) 530 nm, d) 630 nm, e) 830 nm, f) 1030 nm.

The I-T curve data as shown in Figure S8, the Cu:KTN crystal can still achieve self-powered at 250-1030 nm, and the self-powered responsivity are 0.021 mA/W, 0.013 mA/W, 0.006 mA/W, 0.750 μA/W, and 0.145 μA/W, respectively. We find that the response range of KTN after Cu^+^ doping extends to 1030nm, covering the entire visible band and extending to the near infrared region. In the range of 250 nm to 1030 nm, the responsivity of Cu:KTN crystal at 0 V bias voltage decreases with the increase of wavelength, from 5.23 mA/W to 0.145 μA/W.

**1.9 The response time of Cu:KTN to different light sources**


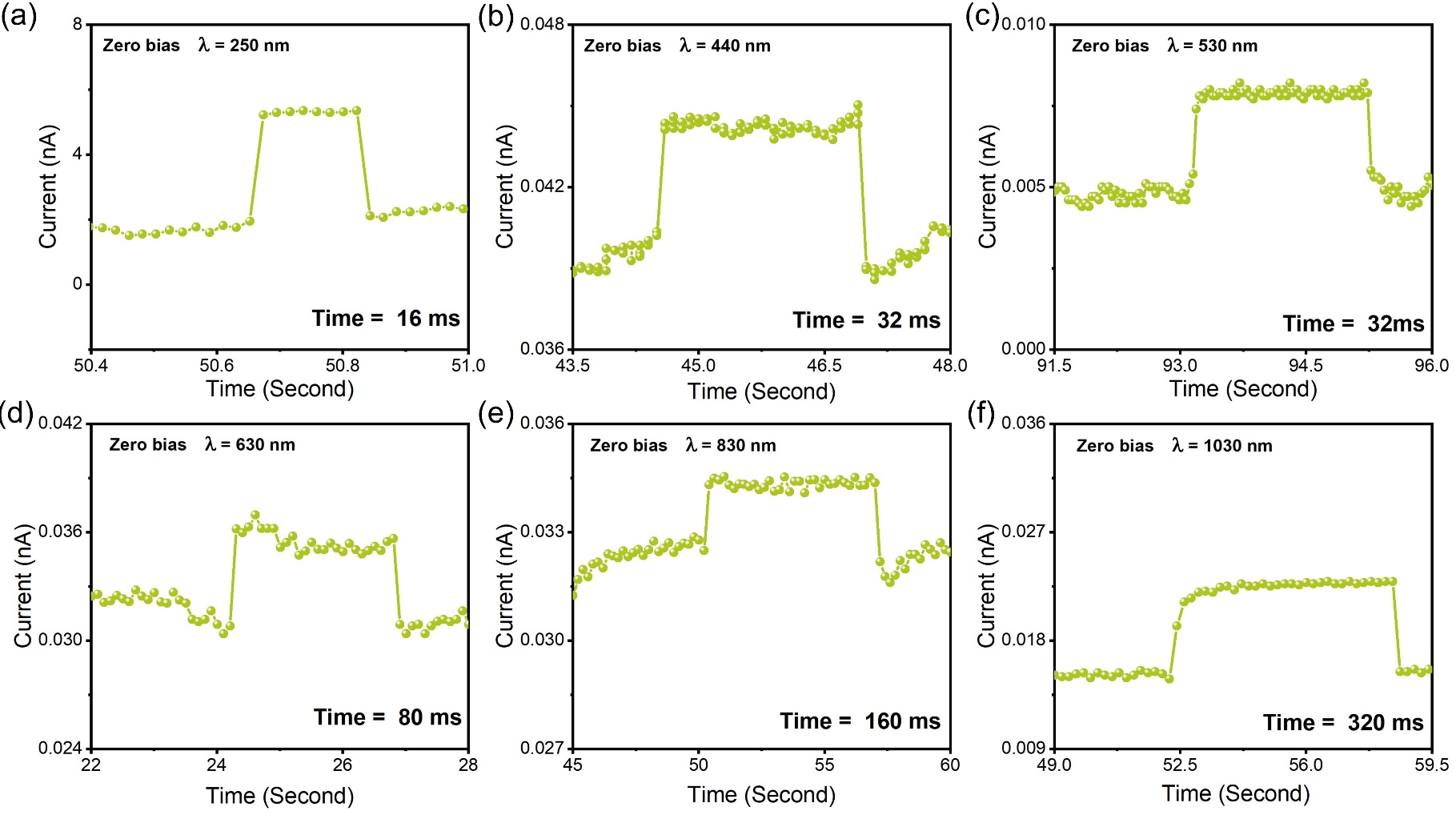


**Figure S9.** The response time of the Cu:KTN based photodetector at a) 250 nm, b) 440 nm, c) 530 nm, d) 630 nm, e) 830 nm, f) 1030 nm.

Response time τ is used to measure the response speed, defined as the time required for the response value to rise from 10% of the peak to 90%. The shorter the response time τ, the faster the device responds to light. We tested the response time τ of Cu:KTN crystal to light sources in different bands and compared it. The response time of Cu:KTN crystal in six bands are 16 ms, 32 ms, 32 ms, 80 ms, 160 ms, 320 ms, respectively. The response time tends to increase with increasing wavelength. We think that the difference in the response time of Cu:KTN crystals to different wavebands is due to the difference in their absorption capacity to different wavelength light sources.

**1.10 The response of Cu:KTN to different light sources at 1240 nm**


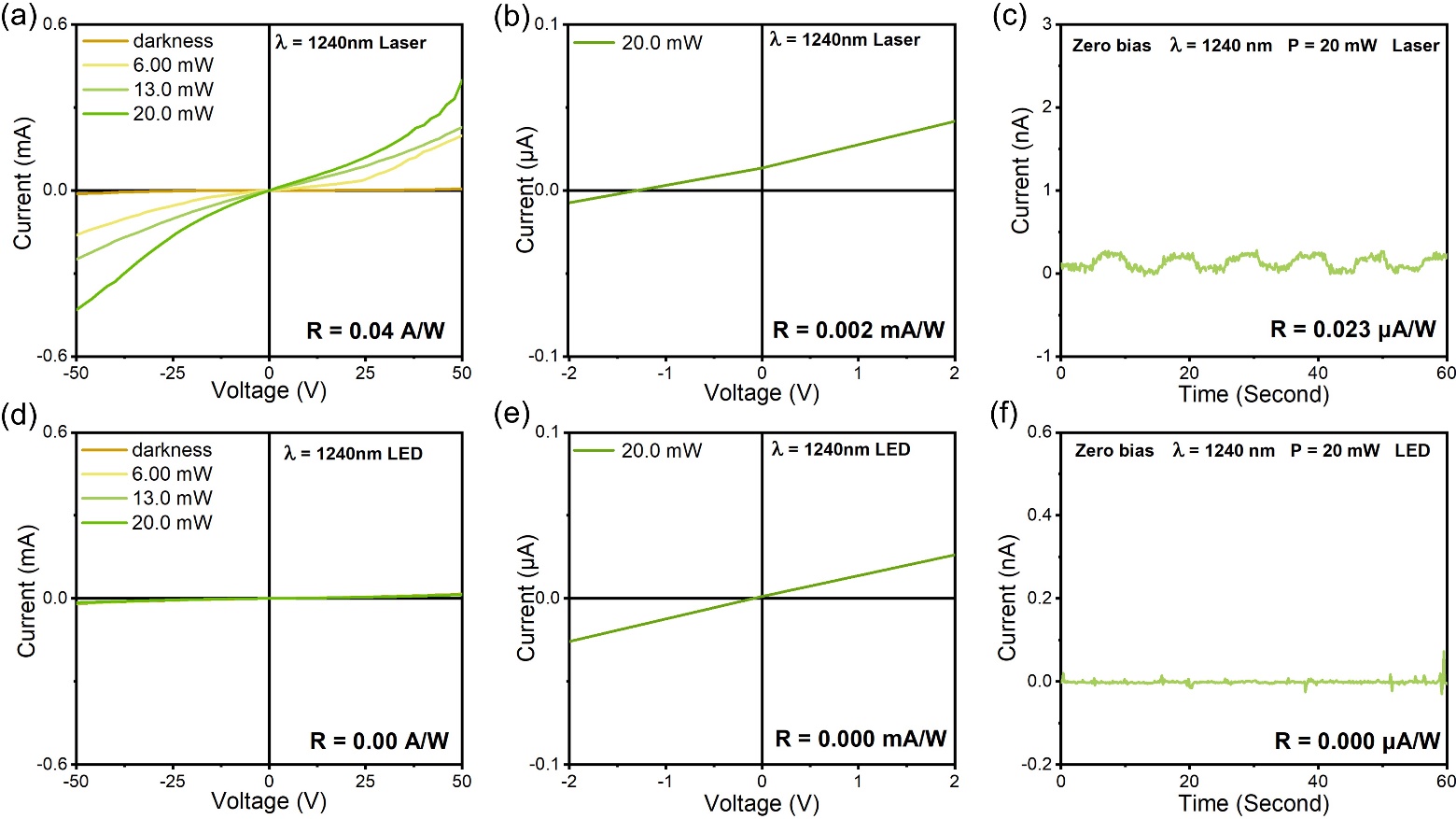


**Figure S10**. a) The current-voltage relationship of the Cu:KTN based photodetector under a 1240 nm Laser. b) The current-voltage relationship of the Cu:KTN based photodetector at low voltage under 1240 nm Laser. c) The current-time relationship of the Cu:KTN based photodetector under a 1240 nm Laser. d) The current-voltage relationship of the Cu:KTN based photodetector under 1240 nm LED. e) The current-voltage relationship of the Cu:KTN based photodetector at low voltage under 1240 nm LED. f) The current-time relationship of the Cu:KTN based photodetector under 1240 nm LED.

To verify whether the frequency doubling effect of KTN crystal can further broaden the response range, a 1240 nm light-emitting diode and a femtosecond pulsed laser were used to incident the Cu:KTN crystal respectively. The current-voltage (I-V) and current-time (I-T) curves are shown in Figure S10. The results show that the Cu:KTN crystal does not respond to the former, which we think is because the Cu:KTN crystal does not absorb light at 1240 nm. However, when the 1240 nm laser is incident, the Cu:KTN crystal produces a response, and we think the reason is that the Cu:KTN crystal produces a 620 nm laser through the frequency doubling effect, and 620 nm is in the absorption range of Cu:KTN. This result validates the feasibility of our idea. In theory, we can use the second harmonic effect of KTN to further expand the response range of KTN crystals to near 2000 nm on the basis of doping Cu.

**1.11 Photocurrent of Cu:KTN crystals at various temperatures**

**
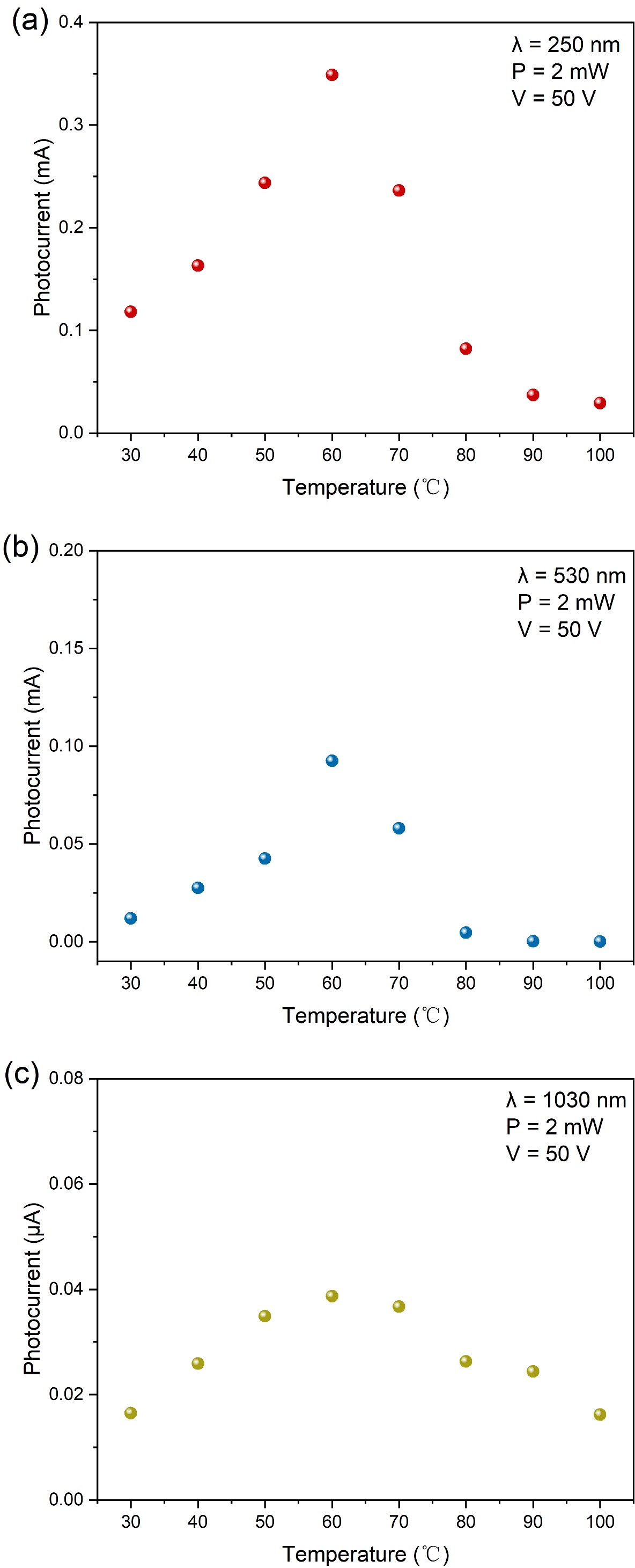
**

**Figure S11**. Dependence of Cu:KTN crystals photocurrent on temperature at a) 250 nm, b) 530 nm, c) 1030 nm.

In order to further study the relationship between photocurrent of Cu:KTN crystals and temperature, we draw the photocurrent-temperature scatter plot (Figure S11). It is found that the photocurrent of the crystal shows the same trend with temperature at different wavelengths. At T<Tc, the photocurrent gradually increases with the temperature rising. When the temperature rises to Tc, the photocurrent reaches maximum. Since the temperature continues to rise, the photocurrent decreases with the increasing temperatures.

**1.12 The I-T curves of the as-grown and poled Cu:KTN crystals**


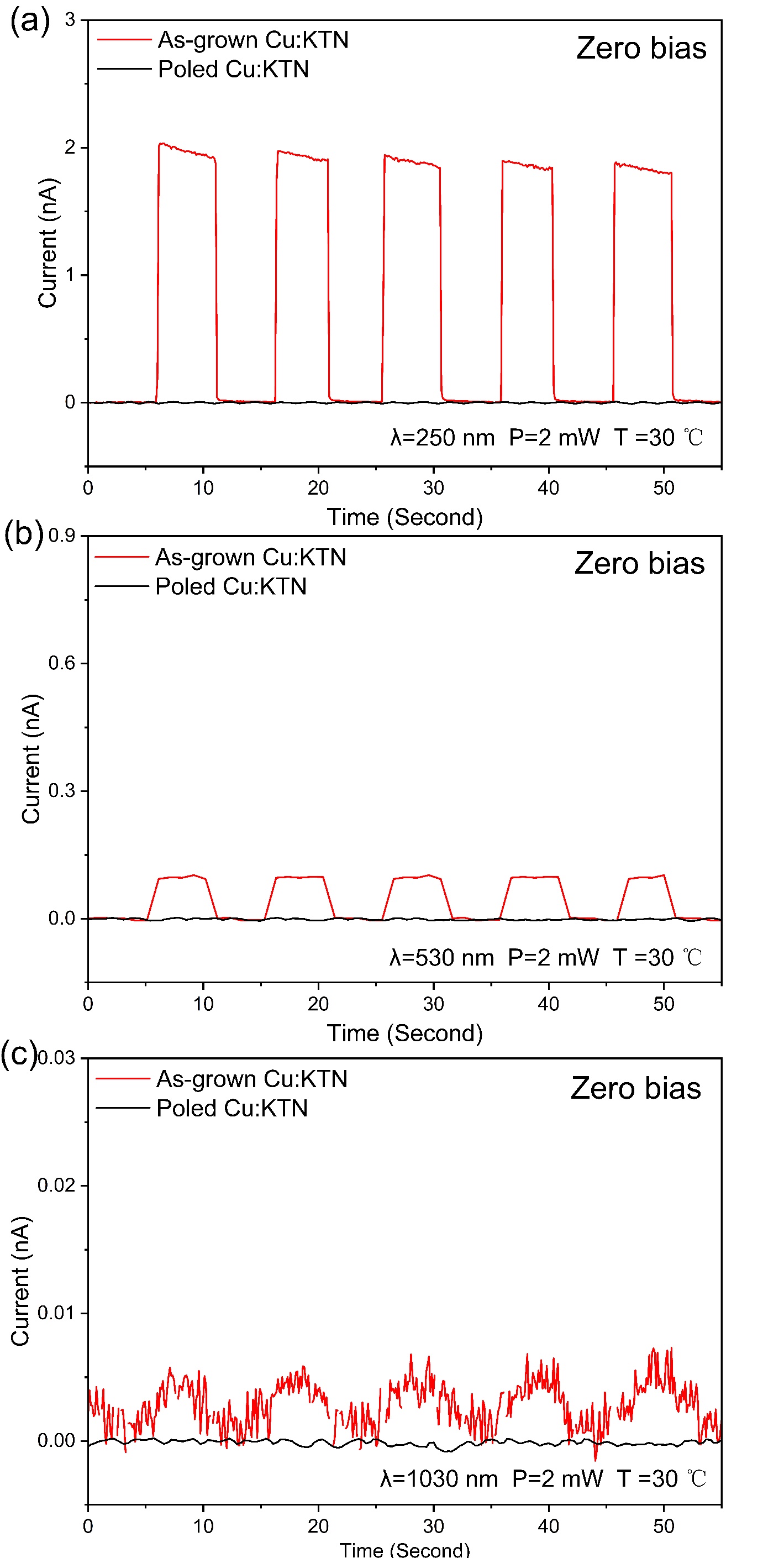


**Figure S12.** The I-T curves of the as-grown and poled Cu:KTN crystals at a) 250 nm, b) 530 nm, and c) 1030 nm

To further explore the effect of ferroelectric domains on the photoresponse, we prepared two Cu:KTN crystals with the same size (4*8*2 mm^3^), and one of them was single-domain poled by an alternating current electric field. Then, the Current-Time (I-T) curves of the Cu:KTN crystals before and after the poling treatment were tested and compared at different wavelengths.

The photocurrent of the poled Cu:KTN crystals decreases significantly at the three wavelengths. At 50 V bias, the comparison of photocurrent between the single-domain Cu:KTN crystal and the as-grown Cu:KTN crystal shows a higher photocurrent more than three orders of magnitude. At 0 V bias, the current of poled crystal before and after illumination does not change and approaches zero. This could be assigned to the disappeared ferroelectric supercells in the poled Cu:KTN crystal. Therefore, this further demonstrates that the photoelectric response of Cu:KTN self-powered detector is dependent on the exotic ferroelectric-orders with ‘head-to-head’ and ‘tail to-tail’ ferroelectric domains.

**1.13 The response of Cu:KTN with different doping concentrations**


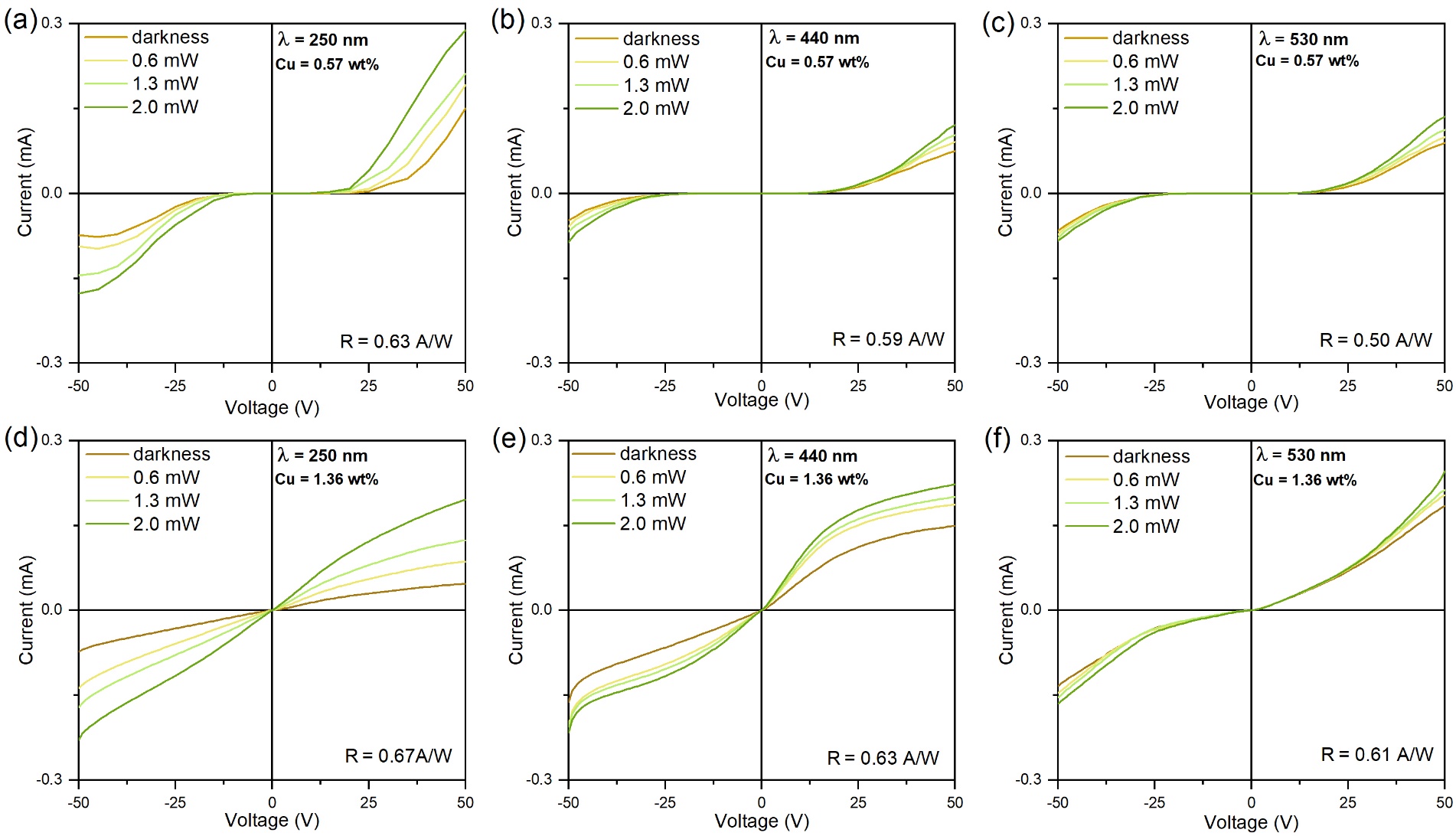


**Figure S13.** Comparison of current-voltage (I-V) curves of Cu:KTN crystals with different doping concentrations at 250 nm, 440nm, and 530 nm.

To explore the effect of Cu^+^ ion doping concentration on the photoelectric performances of Cu:KTN crystals, we grew two Cu:KTN samples with different doping concentrations (0.57 wt% and 1.36 wt%). Gold finger electrodes (all parameters of the electrodes are identical) were sputtered on their surfaces by the magnetron sputtering method and named Cu_0.57_:KTN and Cu_1.36_:KTN. Then we tested the I-V curves of two Cu:KTN samples at 250 nm, 440 nm, and 530 nm.

As shown in Figure S13, when the external voltage is 50 V and the incident light intensity is 2 mW, the responsivity of Cu_0.57_: KTN is 0.63 A/W, 0.59 A/W, 0.50 A/W at 250 nm, 440 nm, and 530 nm, respectively. In contrast, the responsivity of Cu_1.36_:KTN is 0.67 A/W, 0.63 A/W and 0.61 A/W, respectively. Both two Cu:KTN samples exhibited a high optical responsivity and the responsivity gradually decreased at long wavelength. At the same wavelength, the responsivity of Cu_1.36_:KTN is slightly higher than that of Cu_0.57_:KTN, indicating high doping concentration is favorable to improve the photo-responsivity of Cu:KTN detector.

**1.14 The response of Cu:KTN crystals with different electrodes**


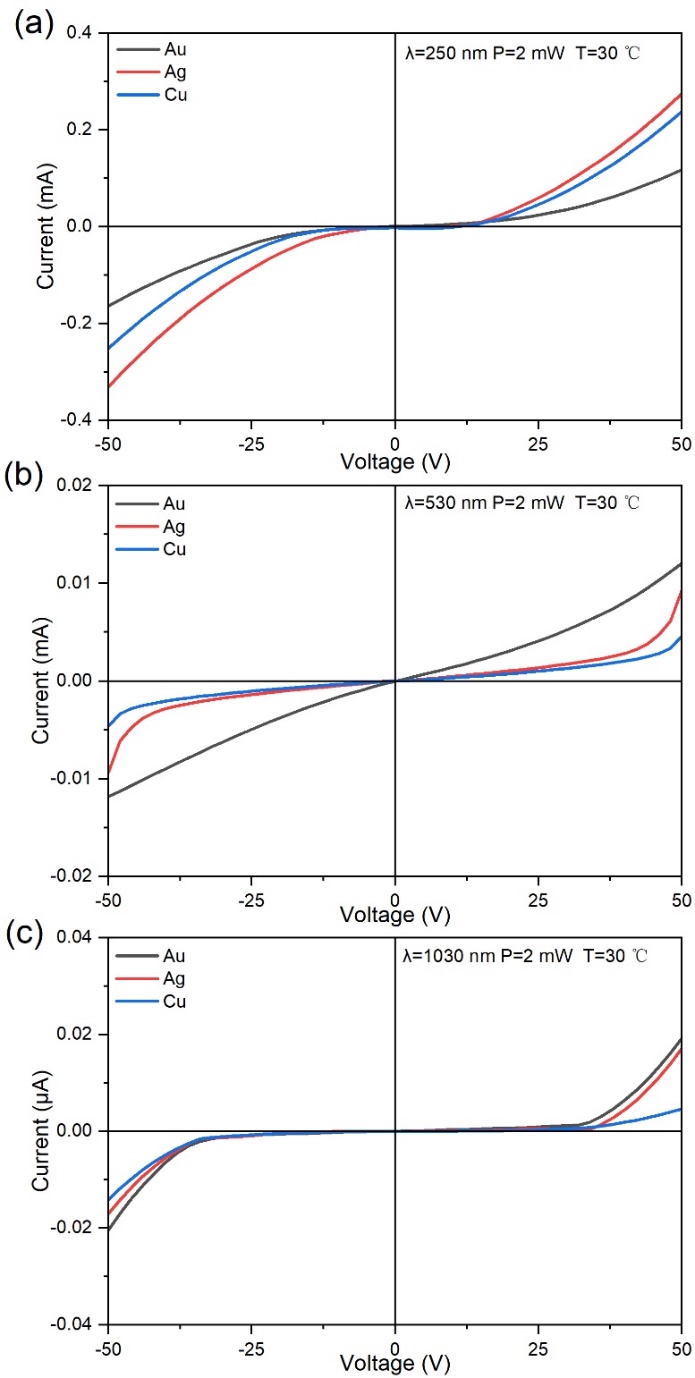


**Figure S14.** The current-voltage (I-V) curves of Cu:KTN crystals with different electrodes at a) 250 nm, b) 530 nm, and c) 1030 nm.

To explore the influence of electrodes on the photoelectric response of Cu:KTN crystals, we prepared three identical Cu:KTN detectors with sputtering Au, Ag, and Cu interdigital electrodes on their surfaces by the magnetron sputtering method. Their photoelectric response was measured at 250 nm, 530 nm, and 1030 nm, respectively.

As shown in Figure S14, all three Cu:KTN detectors show a nonlinear I-V relationship, indicating the photoelectrical performance could not be influenced by electrode interface barriers. The photocurrent of Cu:KTN detector with Au-, Ag-, Cu-electrode is comparable (Table S1), also precluding the influence of electrode interface barriers. When the incident light intensity and test temperature are constant, the photocurrent of Cu:KTN crystal still gradually weakens with the increase of wavelength. At 250 nm, the crystal with Ag-electrode has the highest photocurrent, followed by the Cu-electrode and the Au-electrode. At 530 nm and 1030 nm, the current of Au-electrode is the strongest, the Ag-electrode is the second, and the Cu-electrode is the weakest. We found that in the same band, the photocurrent of the Cu:KTN crystal under the electrodes of the three materials has a certain difference, but the overall gap is small, and the photoelectric response of the crystal does not show a dependence on the electrode material within a reasonable fluctuation range.

**1.15 Photocurrent of Cu:KTN with different electrodes at 50V bias**

**Table S1.** Photocurrent of Cu:KTN crystals with different electrodes at 50V bias

| Electrode | Photocurrent at  250 nm (mA) | Photocurrent at  530 nm (mA) | Photocurrent at  1030 nm (μA) |
| --- | --- | --- | --- |
| Au | 0.12 | 0.012 | 0.019 |
| Ag | 0.28 | 0.009 | 0.017 |
| Cu | 0.24 | 0.005 | 0.005 |

**1.16 The ICP test results of Cu:KTN crystal**

**Table S2.** ICP test results of Cu:KTN crystal

| Sample | Sample Mass  m_0_（g) | Constant Volume  V_0_（mL) | Test Element | Test solution  Element Concentration  C_o_（mg/L) | Dilution Ratio | E lement Concentration  C_1_（mg/L) | Element Content  C_x_（mg/kg) | Element Content  W（%) | Component |
| --- | --- | --- | --- | --- | --- | --- | --- | --- | --- |
| Cu:KTN | 0.0555 | 25 | Cu | 1.264 | 10 | 12.6388434 | 5693.2 | 0.57% | Cu content  0.57wt% |
|  | 0.0555 | 25 | Cu | 1.291 | 10 | 12.9146303 | 5817.4 | 0.58% |  |
|  | 0.0555 | 25 | Nb | 6.883 | 100 | 688.2887314 | 310040.0 | 31.00% | Ta/Nb  0.56/0.44 |
|  | 0.0555 | 25 | Nb | 6.872 | 100 | 687.2491555 | 309571.7 | 30.96% |  |
|  | 0.0555 | 25 | Ta | 1.513 | 1000 | 1512.833567 | 681456.6 | 68.15% |  |
|  | 0.0555 | 25 | Ta | 1.515 | 1000 | 1514.845032 | 682362.6 | 68.24% |  |

The inductively coupled plasma test is obtained by ICP-OES spectrometer (Agilent 5110), test elements include Cu, Ta, and Nb. The test results give a Cu content of 0.57 wt% and a Ta/Nb ratio of 0.56/0.44, which is consistent with the result of XPS in the main text Figure 2c.

**1.17 Photocurrent of Cu:KTN at various temperatures**

**Table S3.** Photocurrent of Cu:KTN crystals at various temperatures with 50 V bias

| Temperature | Photocurrent at  250 nm (mA) | Photocurrent at  530 nm (mA) | Photocurrent at  1030 nm (μA) |
| --- | --- | --- | --- |
| 30 ℃ | 0.1182 | 0.0120 | 0.0165 |
| 40 ℃ | 0.1631 | 0.0276 | 0.0259 |
| 50 ℃ | 0.2436 | 0.0426 | 0.0349 |
| 60 ℃ | 0.3489 | 0.0925 | 0.0387 |
| 70 ℃ | 0.2362 | 0.0580 | 0.0367 |
| 80 ℃ | 0.0822 | 0.0046 | 0.0263 |
| 90 ℃ | 0.0371 | 0.0003 | 0.0244 |
| 100 ℃ | 0.0293 | 0.0002 | 0.0162 |

**1.18 Comparison of self-powered responsivity of common ferroelectric materials**

**Table S4.** Comparison of self-powered responsivity of common ferroelectric materials

| Ferroelectric material | Wavelength  (nm) | Power density  (mW/cm^2^) | Net current  at zero bias  (nA) | Responsivity  (mA/W) | References |
| --- | --- | --- | --- | --- | --- |
| BaTiO_3_  (BTO) | 405 | 100 | 0.2 | 2×10^-6^ | [8] |
| KH_2_PO_4_  (KDP) | 300 | 0.1 | 3×10^-4^ | 3×10^-6^ | [9] |
| [KNbO_3_]_1-X_[BaNi_1/2_Nb_1/2_O_3&_]_X_  (KBNNO) | 700 | 4.0 | 5×10^-4^ | 9×10^-6^ | [10] |
| Fe:KTa_0.41_Nb_0.59_O_3_  (Fe:KTN59) | 405 | 100 mW | 18.5 | 1.85×10^-4^ | [11] |
| BA_2_CsPb_2_Br_7_  (BACPB) | 405 | 37.06 | 8×10^-3^ | 2×10^-4^ | [12] |
| EA_4_Pb_3_Cl_10_  (EAPC) | 206 | 7.09 mW/mm^2^ | 18.6 μA/cm^2^ | 2.62×10^-4^ | [13] |
| Bi_0.5_Na_0.5_TiO_3_  (BNT) | 365 | 85.2 | 1.2×10^3^ | 1.4×10^-2^ | [14] |
| KTa_0.59_Nb_0.41_O_3_  （KTN41） | 280 | 3.0 | 200 | 0.11 | [15] |
| (isopentylammonium)_2_(ethylammonium)_2_Pb_3_I_10_ | 637 | 127 mW/cm^2^ | 1.5 μA/cm^2^ | 0.01 | [16] |
| (EA)_2_(MA)_2_Pb_3_Br_10_ | 405 | 0.1 W/cm^2^ | 4.16 | 4.16×10^-5^ | [17] |
| Cu:KTa_0.56_Nb_0.44_O_3_  (Cu:KTN44) | 250 | 19.9 | 32.6 | 5.11 | **This work** |

**1.19 Comparison of photo-responsive ranges of common ferroelectric materials**

**Table S5.** Comparison of response ranges of common ferroelectric materials

| Ferroelectric material | Responsive range  (nm) | Temperature | Reference |
| --- | --- | --- | --- |
| Cu:KTa_0.56_Nb_0.44_O_3_  (Cu:KTN44) | 250-1030 | Room temperature | **This work** |
| [KNbO_3_]_1-X_[BaNi_1/2_Nb_1/2_O_3&_]_X_  (KBNNO) | 700 |  | [10] |
| BiFeO_3_ | 450-650 |  | [18] |
| AgNbO_3_ | 405-550 |  | [19] |
| Fe:KTa_0.41_Nb_0.59_O_3_  (Fe:KTN59) | 405 |  | [11] |
| Fe：BaTiO_3_ | 400-700 |  | [20] |
| BaTiO_3_  (BTO) | 375-405 |  | [8] |
| Bi_4_Ti_3_O_12_ | 375-405 |  | [21] |
| KH_2_PO_4_  (KDP) | 300 |  | [9] |
| KTa_0.59_Nb_0.41_O_3_  （KTN41） | 280 |  | [15] |

**References**

[1] Y. Wang, I. Di Sarcina, A. Cemmi, S. Baccaro,G. Chen, *Opt. Mater.* **2019**, *87*, 80.

[2] H. Gu, C. Song, Y. Cui, *J.* *Lumin.* **2010**, *130*, 78.

[3] T. Lv, X. Xu, X. Yu, H. Yu, D. Zhou, J. Qiu, *J. Am. Chem. Soc.* **2014**, *97*, 2897.

[4] D. Wang, J. Hlinka, A. A. Bokov, Z. Ye, P. Ondrejkovic, J. Petzelt, L. Bellaiche, *Nat. Commun*. **2014**, 5, 5100.

[5] L. Ding, E. Beyreuther, B. Koppitz, K. Kempf, J. Ren, W. Chen, M. Rusing, Y. Zheng, L. Eng, Appl. Phys. Lett. **2024**, 124, 252901.

[6] Y. Sun, Z. Zhang, X. Fang, Z. Huang, D. Zhou, H. Tian, G. Han, Z. Ren, APL Mater. **2023**, 11, 091116.

[7] Y. Yun, L. Muhlenbein, D. S. Knoche, A. Lotnyk, A. Bhatnagar, *Sci. Adv.* **2021**, 7, eabe4206.

[8] Y. Pu, M. Yao, H. Liu, T. Frömling, *J. Eur. Ceram. Soc.* **2016***,* *36****,*** 2461-2468.

[9] S. Van Aert, S. Turner, R. Delville, D. Schryvers, G. Van Tendeloo, E. K. Salje, *Adv. Mater.* **2012***, 24****,*** 523.

[10] G. Nataf, M. Guennou, J. Kreisel, P. Hicher, R. Haumont, O. Aktas, E. Salje, L. Tortech, C. Mathieu, D. Martinotti, *Phys. Rev. Mater.* **2017***, 1,* 074410.

[11] J. Seidel, L. W. Martin, Q. He, Q. Zhan, Y. H. Chu, A. Rother, M. E. Hawkridge, P. Maksymovych, P. Yu, M. Gajek, *Nat. Mater.* **2009***, 8****,*** 229-234.

[12] J. Wang, Y. Liu, S. Han, Y. Ma, Y. Li, Z. Xu, J. Luo, M. Hong, Z. Sun, *Sci. Bull.* **2021***, 66****,*** 158-163.

[13] X. Liu, S. Wang, P. Long, L. Li, Y. Peng, Z. Xu, S. Han, Z. Sun, M. Hong, J. Luo, *Angew. Chem. Int. Ed.* **2019***, 131****,*** 14646-14650.

[14] Z. L. Wang, *Sci. Am.* **2008***, 298****,*** 82-87.

[15] Y. Wu, X. Wang, G. Tian, L. Zheng, F. Liang, S. Zhang, H. Yu, H. Zhang, *Adv. Mater.* **2022***, 34****,*** 2105108.

[16] S. Han, M. Li, Y. Liu, W. Guo, M. Hong, Z. Hong, J. Luo, *Nat. Commun.* **2021***,* *12****,*** 284.

[17] X. Liu, S. Wang, P. Long, L. Li, Y. Peng, Z. Xu, S. Han, Z. Sun, M. Hong, J. Luo, *Angew.Chem.Int. Ed.* **2019**, *58*,14504.

[18] Z. Li, Y. Zhao, W.-L. Li, R. Song, W. Zhao, Z. Wang, Y. Peng, W.-D. Fei, *J. Phys. Chem. C* **2021***, 125****,*** 9411-9418.

[19] X. He, C. Chen, C. Li, H. Zeng, Z. Yi, *Adv. Funct. Mater.* **2019***, 29****,*** 1900918.

[20] Y. Noguchi, Y. Taniguchi, R. Inoue, M. Miyayama, *Nat. Commun.* **2020***,* *11****,*** 966.

[21] Y. Yuan, Z. Xiao, B. Yang, J. Huang, *J. Mater. Chem. A* **2014***, 2****,*** 6027-6041.
